# The GLYT1 inhibitor bitopertin mitigates erythroid PPIX production and liver disease in erythroid protoporphyria

**DOI:** 10.1172/JCI181875

**Published:** 2025-07-15

**Authors:** Sarah Ducamp, Min Wu, Juan Putra, Dean R. Campagna, Yi Xiang, Vu Hong, Matthew M. Heeney, Amy K. Dickey, Rebecca K. Leaf, Mark D. Fleming, Brian MacDonald, Paul J. Schmidt

**Affiliations:** 1 Department of Pathology, Boston Children’s Hospital and Harvard Medical School, Boston, Massachusetts, USA.; 2 Disc Medicine, Watertown, Massachusetts, USA.; 3 Dana-Farber Boston Children’s Cancer and Blood Disorders Center and Department of Pediatrics, Harvard Medical School, Boston, Massachusetts, USA.; 4 Division of Pulmonary and Critical Care Medicine, Department of Medicine, and; 5 Division of Hematology/Oncology, Massachusetts General Hospital and Harvard Medical School Boston, Massachusetts, USA

**Keywords:** Genetics, Hematology, Genetic diseases

## Abstract

Erythropoietic protoporphyria (EPP) is a genetic disorder typically resulting from decreased ferrochelatase (FECH) activity, the last enzyme in heme biosynthesis. Patients with X-linked protoporphyria (XLPP) have an overlapping phenotype caused by increased activity of 5-aminolevulinic acid synthase 2 (ALAS2), the first enzyme in erythroid heme synthesis. In both cases, protoporphyrin IX (PPIX) accumulates in erythrocytes and secondarily in plasma and tissues. Patients develop acute phototoxicity reactions upon brief exposure to sunlight. Some also experience chronic liver disease, and a small fraction develop acute cholestatic liver failure. Therapeutic options are limited, and none, save hematopoietic stem cell transplantation, directly targets erythroid PPIX accumulation. Bitopertin is an investigational orally available small-molecule inhibitor of the erythroid cell-surface glycine transporter GLYT1. We established the bitopertin PPIX inhibitory half-maximal effective concentration in a human erythroblast EPP model and confirmed a marked reduction of PPIX in erythroblasts derived from patients with EPP. We demonstrate that bitopertin also reduced erythrocyte and plasma PPIX accumulation in vivo in both EPP and XLPP mouse models. Finally, the reduction in erythroid PPIX ameliorated liver disease in the EPP mouse model. Altogether, these data support the development of bitopertin to treat patients with EPP or XLPP.

## Abstract 2

This manuscript describes a the successful use of the oral glycine uptake inhibitor to treat a mouse model of erythropoietic protoporphyria.

## Graphical abstract

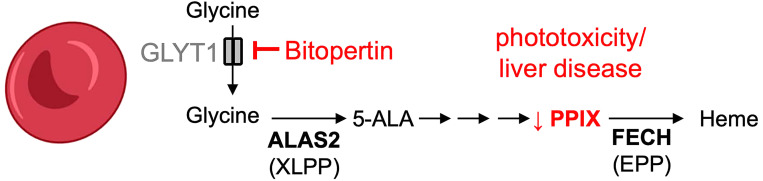

## Introduction

**Figure 1:**
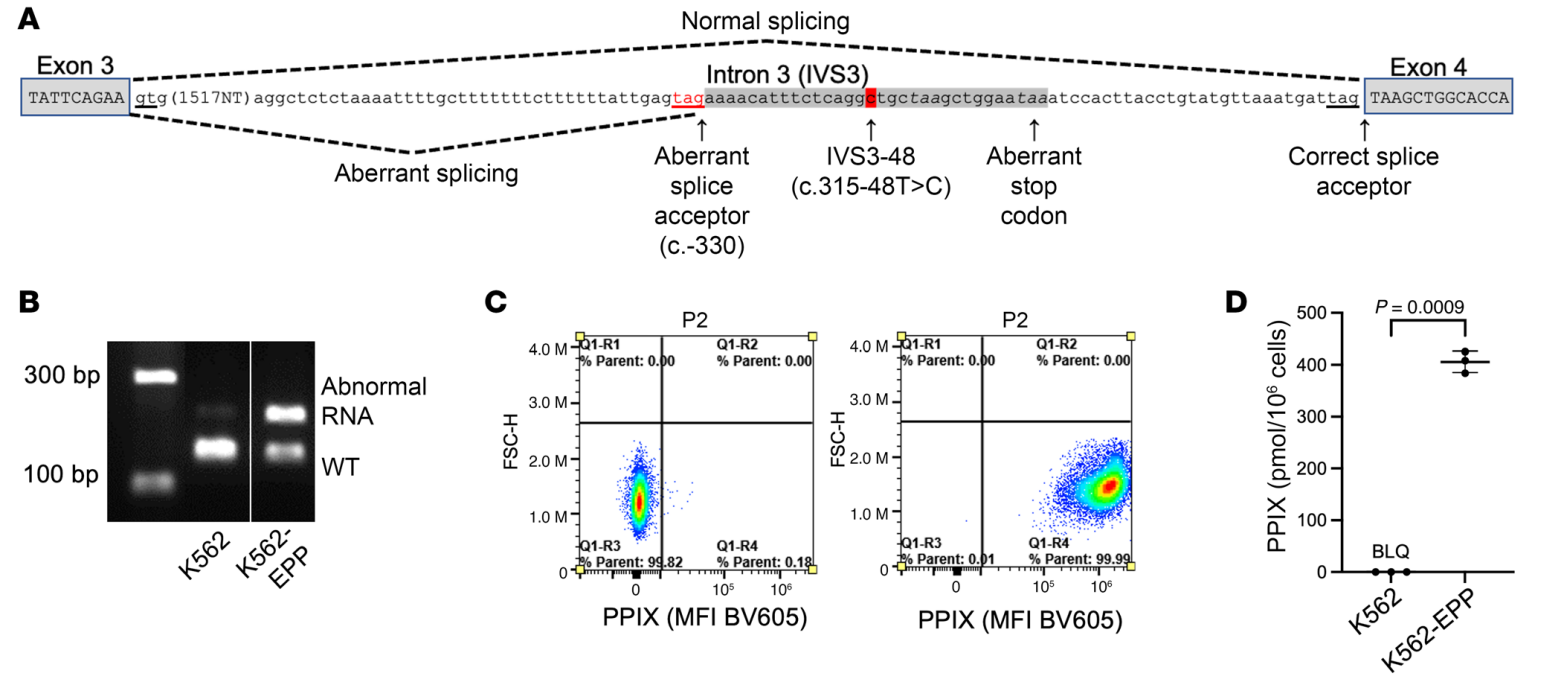
Generation of a K562 human erythroleukemic cell line with EPP. (**A**) Schematic of the human *FECH* locus showing the c.315-48T>C low expression splice variant (adapted from Mirmiran et al., ref. 20). (**B**) Aberrant *FECH* splicing in the K562-EPP cell line model of EPP. Lanes were run on the same gel but were noncontiguous, as indicated by the white line. (**C**) PPIX fluorescence in the control K562 (left) and K562-EPP (right) cell lines. FSC-H, forward scatter – height. (**D**) Quantification of PPIX levels in control K562 and K562-EPP cell lines. BLQ, below the limit of quantification. *P* values were calculated using an unpaired, 2-tailed *t* test with Welch’s correction. Data are presented as the mean ± SD.

Erythropoietic protoporphyria (EPP) (OMIM: 177000) is a cutaneous light sensitivity disorder that typically results from pathogenic variants in ferrochelatase (FECH), the last enzyme in heme biosynthesis. A decrease of more than approximately 65% of FECH activity causes accumulation of the photoactive pigment metal-free protoporphyrin IX (PPIX) in erythrocytes and secondarily in plasma and tissues (1). Approximately 95% of patients with EPP have a rare, pathogenic missense, nonsense, spliced, or deleted *FECH* allele in *trans* of a common intronic variant c.315-48T>C (rs2272783) that increases the use of an aberrant splice site, resulting in a premature stop codon (Figure 1A).

X-linked protoporphyria (XLPP) (OMIM #300752) is largely clinically indistinguishable from EPP (2, 3). Patients with XLPP have C-terminal truncating pathogenic variants in the last exon of 5-aminolevulinic acid synthase 2 (ALAS2), the first and rate-limiting step in erythroid heme synthesis. ALAS2 condenses glycine with succinyl coenzyme A to produce 5-ALA (ALA), which is subsequently multimerized and modified to form PPIX. XLPP ALAS2 pathogenic variants lead to the loss of a negative regulatory domain, resulting in ALAS2 gain-of-function alleles that have increased catalytic activity, leading to ALA overproduction and PPIX accumulation. XLPP and EPP are collectively called protoporphyria, and less than 10% of patients with protoporphyria have XLPP (4, 5).

The prevalence of EPP and XLPP in Europe is reported to be approximately 1:100,000 individuals (6). An analysis of FECH variants in the UK Biobank has provided evidence that EPP prevalence is 1:17,000 (5, 7). Patients with protoporphyria experience life-long, painful phototoxicity from visible light exposure (1). Phototoxicity manifests as severe pain episodes that last for days and are unresponsive to analgesics, including narcotics. Even casual exposure to sunlight can precipitate pain. Moreover, due to light sensitivity, patients often have limited career and social opportunities that significantly affect their quality of life (8, 9).

In addition to photosensitivity, patients may also develop anemia, gallstones, and liver disease. Whereas 20%–30% of patients have chronic hepatic transaminitis, 1%–5% of patients will develop acute cholestatic liver failure that can be fatal or require liver transplantation (10, 11). A liver transplant may be followed by hematopoietic stem cell transplantation (HSCT) to prevent the recurrence of PPIX-related liver disease in the donor liver (12). Even though HSCT is curative, because of the up-front risk of death and long-term complications, such as graft versus host disease, HSCT is not used as first-line therapy.

Light sensitivity in protoporphyria extends into the visible spectrum, and sunscreens do not protect against phototoxic reactions. The only approved drug for phototoxicity prevention in adults is afamelanotide, a subcutaneous implant of a synthetic analog of human α-melanocyte–stimulating hormone, which increases melanin levels in the skin to provide protection against ultraviolet radiation in sunlight (13). Afamelanotide leads to an increase in pain-free light exposure and improvement in quality of life. It has also been hypothesized to improve liver function through an undefined mechanism (13, 14). Other drugs or approaches have been investigated (15, 16). Some, such as isoniazid, were unsuccessful (17, 18). ALAS2 inhibitors (19), an antisense oligonucleotide strategy targeting the c.315-48C allele (20, 21), and several other therapies are currently in development or clinical trials (22–25).

Heme production in the erythroblast is critically dependent on intramitochondrial glycine, which is a substrate for ALAS2. The very high demand for heme in the developing RBC requires extracellular glycine for adequate hemoglobinization. Deletion of the cell-surface glycine transporter (GLYT1), which is expressed primarily in the brain and erythroid precursors, results in decreased fetal liver erythroblast glycine uptake, reduced iron incorporation into heme, and microcytic anemia in newborn mice (26).

Bitopertin is an investigational oral, potent, and selective noncompetitive inhibitor of GLYT1 and has favorable pharmacokinetic properties, including an approximately 40-hour half-life in humans (27–29). The drug was initially developed for treatment of the negative symptoms (e.g., apathy and social withdrawal) of schizophrenia. Although early studies of bitopertin suggested efficacy in schizophrenia, this was not confirmed in later phase III trials (30–33). Nonetheless, bitopertin has exhibited an acceptable safety profile in clinical trials that have altogether enrolled over 4,000 participants.

In healthy volunteers, bitopertin results in a dose-dependent, but clinically negligible, reduction in hemoglobin (Hb) and results in erythrocytes that are hypochromic and microcytic (30, 33–35). Preclinical work in rats demonstrated that chronic dosing resulted in a microcytic, hypochromic, regenerative anemia with siderocytes (36). Treatment of patients with another structurally unrelated GLYT1 inhibitor also induces mild anemia, suggesting that the effect on erythropoiesis is GLYT1 dependent (37). The drug has been shown to decrease erythroid PPIX and heme levels in human healthy and β-thalassemic erythroid precursors (38). We hypothesized that bitopertin might effectively reduce erythroid metal-free PPIX accumulation in protoporphyric cells, potentially resulting in downstream clinical benefits such as decreased photosensitivity and liver disease. During the conduct of the studies reported here, Halloy and collaborators tested several glycine uptake inhibitors for their effect on heme synthesis and demonstrated that high concentrations of bitopertin (1 μM) reduced PPIX accumulation (30%–40%) in erythroblasts derived from CD34^+^ hematopoietic stem cells (HSCs) from a single patient with EPP (39). Bitopertin has now completed phase II clinical trials to evaluate its efficacy in erythropoietic protoporphyria (NCT05308472 and ACTRN12622000799752).

Here, we examined the effects of bitopertin in EPP and XLPP. We established that bitopertin has an EC_50_ of 32 nM in a cellular model of EPP generated by knocking down FECH in erythroblasts. We also confirmed that bitopertin reduced PPIX accumulation in erythroblasts derived from adult HSCs obtained from 3 unrelated patients with EPP, at a final concentration of as low as 10 nM. In mouse models of EPP and XLPP, oral administration of bitopertin reduced erythrocyte PPIX accumulation in a dose-dependent manner and rapidly acted to limit PPIX accumulation in the reticulocytes, which contained disproportionate amounts of PPIX compared with mature RBCs. Treatment with 200 parts per million (ppm) bitopertin for 8 and 32 weeks was associated with improvement of some liver disease parameters, significantly reducing liver fibrosis and ductular reaction in the EPP model. Altogether, these results support the ongoing development of bitopertin as an agent for the treatment of EPP and XLPP.

## Results

### Bitopertin reduces PPIX accumulation in EPP cell models and in EPP patient–derived erythroblasts

#### Effect of bitopertin on K562-EPP cells.

**Figure 2:**
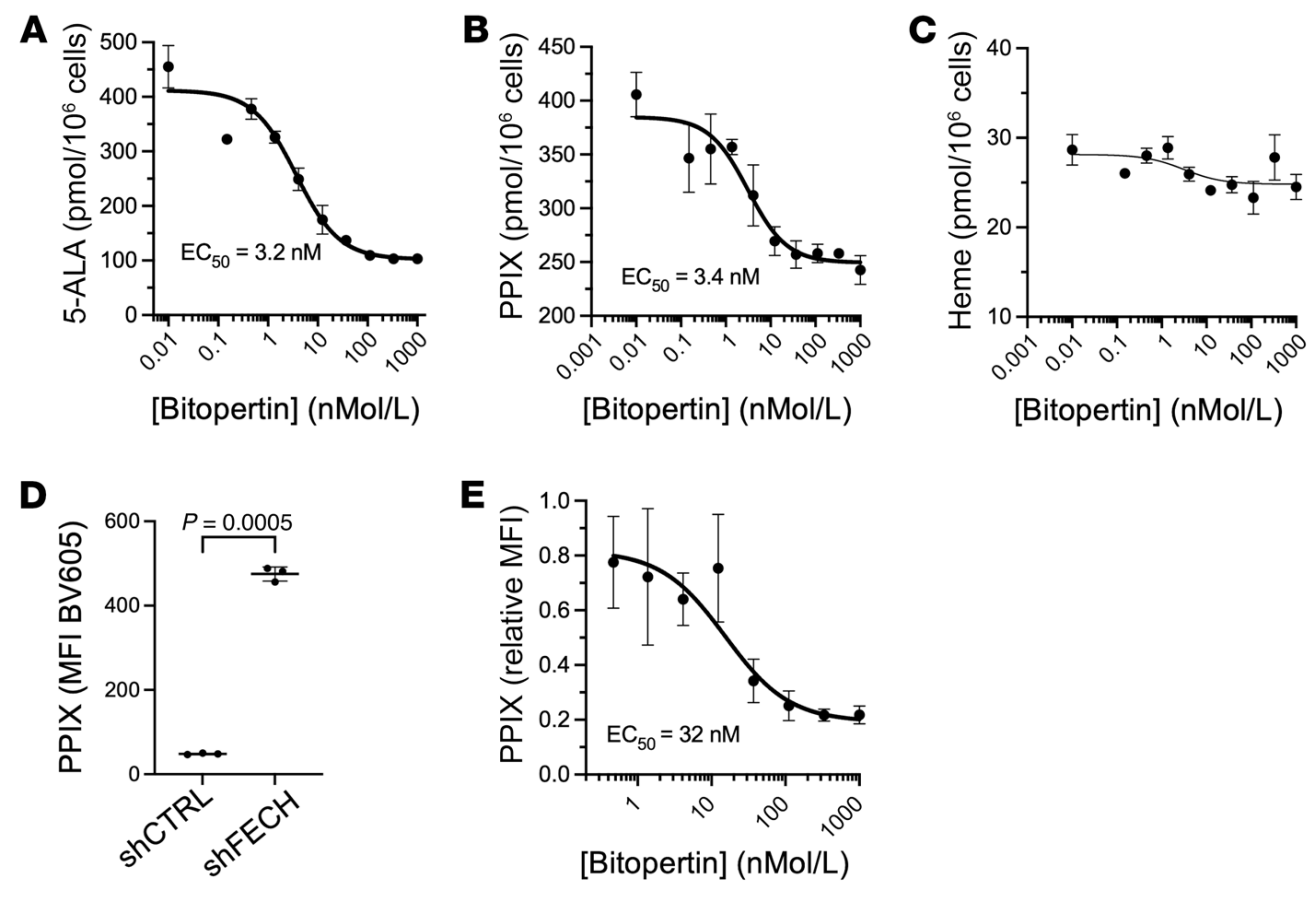
In vitro effects of bitopertin in the K562-EPP cell line and FECH-depleted human erythroblasts. Data showing (**A**) 5-aminolevulinic acid (5-ALA), (**B**) PPIX, and (**C**) heme production in response to varying concentrations of bitopertin in K562-EPP cells. (**D**) PPIX accumulation in human umbilical vein HSCs treated with control or *FECH* shRNAs and differentiated into erythroblasts. *P* values were calculated using an unpaired, 2-tailed *t* test with Welch’s correction. (**E**) Dose response of PPIX production to varying concentrations of bitopertin in human umbilical vein HSCs treated with *FECH* shRNAs differentiated into erythroblasts. Data are presented as the mean ± SD.

To confirm that bitopertin can reduce PPIX accumulation in EPP, we created a K562 erythroid cell line with an EPP genotype (K562-EPP) by introducing the common hypomorphic variant c.315-48T>C in *trans* of a null allele (p.Thr81fs8*). As expected, the c.315-48T>C allele resulted in an aberrant, longer *FECH* mRNA (Figure 1B). The PPIX concentration in undifferentiated K562-EPP cells was more than 10,000-fold higher than in the parental cell line (405.7 ± 20.6 pmol/10^6^ cells vs < 0.2 pmol/10^6^ cells; Figure 1, C and D). In K562-EPP cells, bitopertin inhibited the production of ALA and PPIX in a dose-dependent manner, with EC_50_ values of 3.2 nM and 3.4 nM, respectively (Figure 2, A and B), and had little effect on heme synthesis (Figure 2C).

#### FECH knockdown in human erythroblasts.

We expanded human umbilical cord HSCs and differentiated them into erythroblasts for 16 days after infection with a lentiviral vector encoding either a control shRNA or *FECH* shRNA. This resulted in an approximately 80% decrease in *FECH* mRNA and protein expression levels and in an approximately 10-fold increase in PPIX fluorescence (Supplemental Figure 1, B and C, and Figure 2D; supplemental material available online with this article; https://doi.org/10.1172/JCI181875DS1). In this model, the PPIX inhibitory EC_50_ of bitopertin was 32 nM (Figure 2E). Over a range of concentrations from 0.45 nM to 1 μM, we found that bitopertin had no effect on cell viability or on the fraction of CD235^+^CD71^+^ erythroblasts (Supplemental Figure 1D).

#### EPP patient–derived erythroblasts.

**Figure 3:**
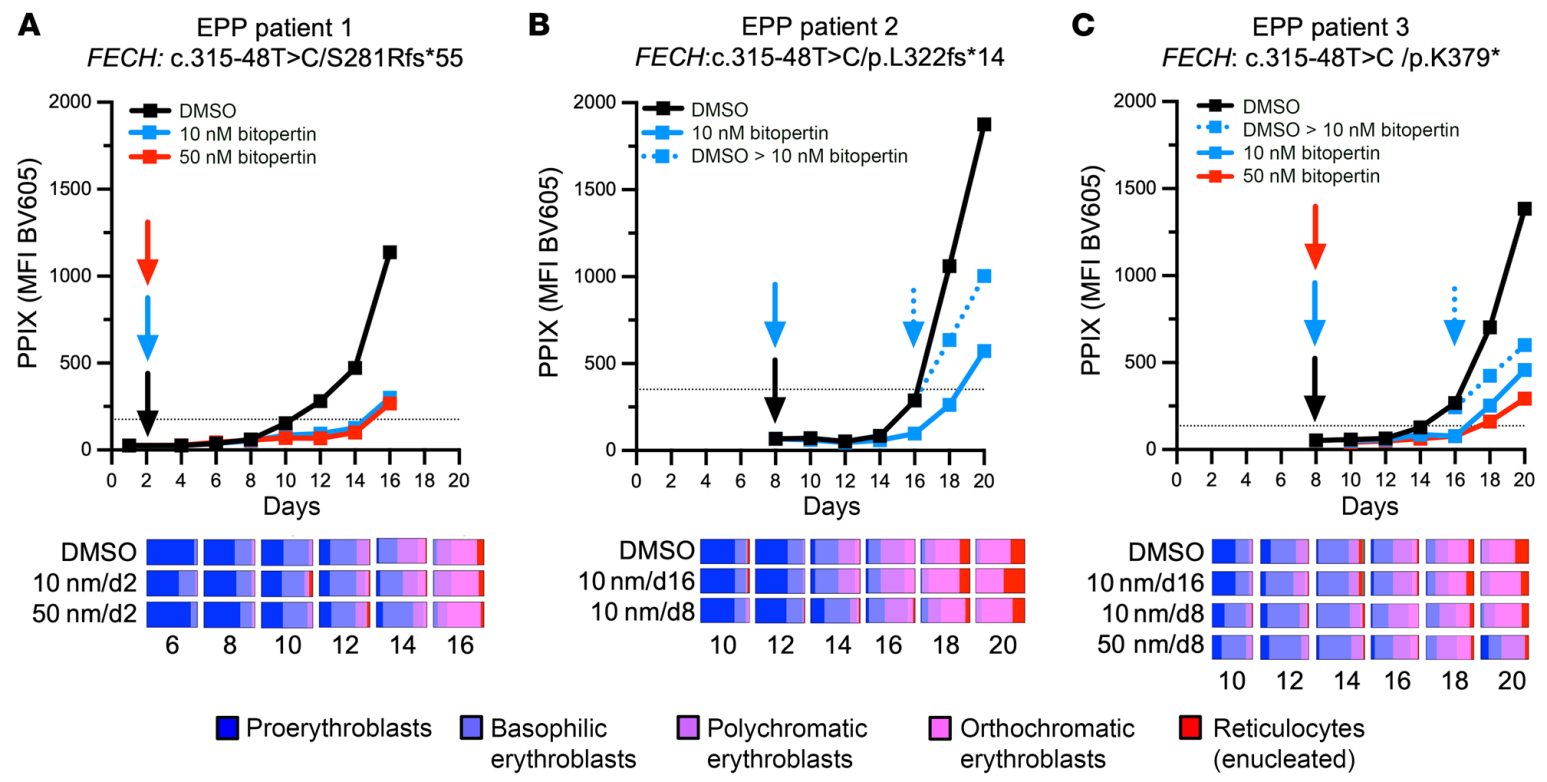
In vitro effects of bitopertin in erythroblasts differentiated from EPP patients. (**A**–**C**) Time course of PPIX accumulation and morphologic analyses in independent differentiation experiments. Erythroblasts were differentiated using PB from 3 unrelated EPP donors (EPP patients 1, 2, and 3) and exposed to DMSO or 10 nM or 50 nM bitopertin in DMSO beginning before or after PPIX accumulation. Horizontal dotted lines indicate the MFI of the PB at the collection of each donor sample.

We isolated CD34^+^ HSCs from the peripheral blood (PB) of 3 unrelated patients with EPP and differentiated each sample into erythroblasts in the presence of vehicle (DMSO) or bitopertin either in the range of the EC_50_ of 10 nM, as previously used in thalassemic erythroblasts (38), or 50 nM (target dose) bitopertin added before the initial accumulation of PPIX (Figure 3, A–C), or 10 nM bitopertin added after the initial accumulation of PPIX (Figure 3, B and C). As a limited number of cells were obtained, only the 10 nM concentration added at the beginning of differentiation was evaluated in all 3 patients. Compared with controls, each bitopertin treatment scheme resulted in a marked reduction in PPIX accumulation, while it had only a limited dose- and time-dependent effect on morphological differentiation.

### Bitopertin inhibits glycine uptake in mouse and rat reticulocytes

**Figure 4:**
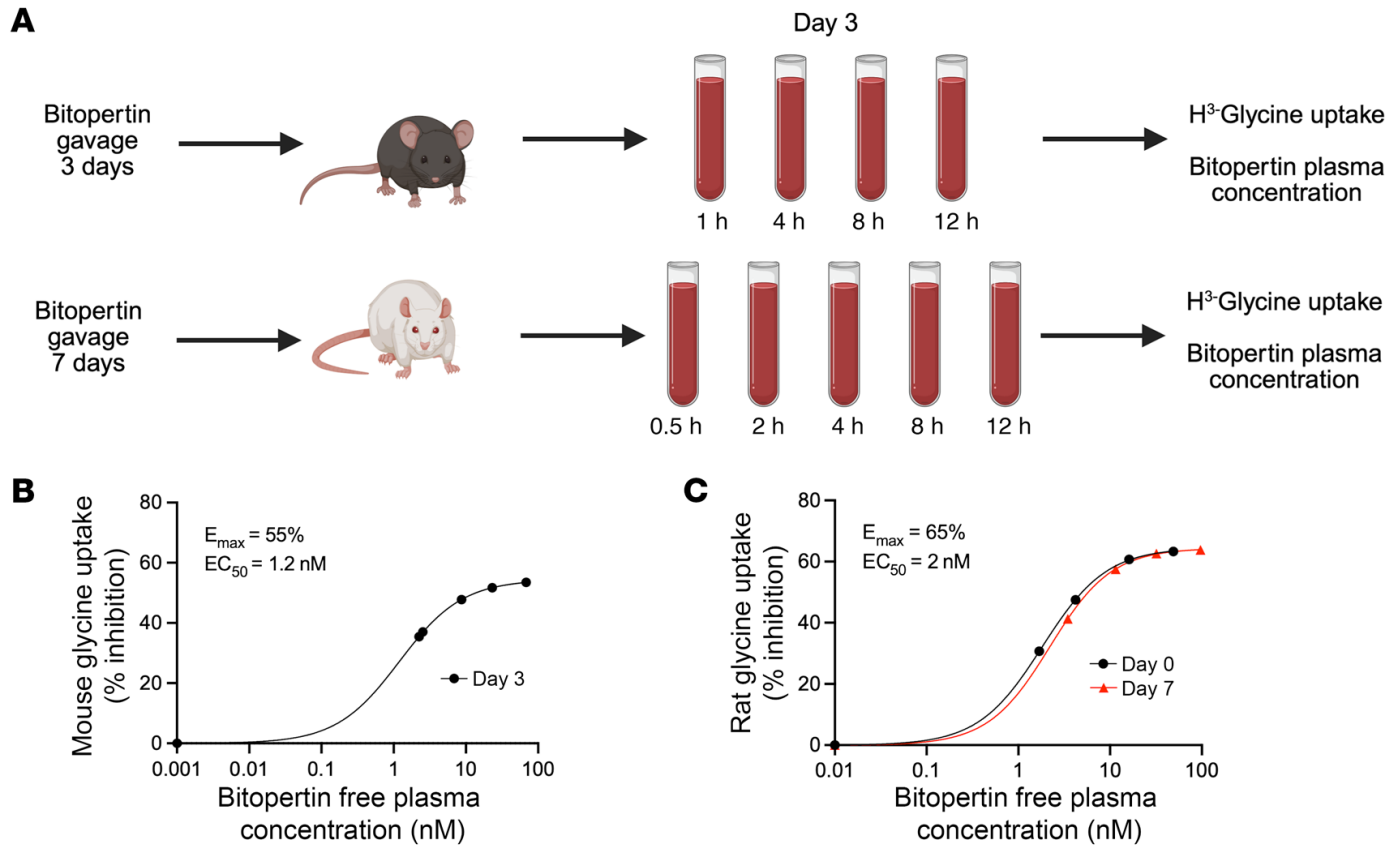
In vitro glycine uptake of rodent PB exposed to bitopertin in vivo. (**A**) Experimental schema. (**B**) Male mice were exposed to daily oral gavage of 0, 0.3, 1, 3, 10, or 30 mg/kg bitopertin once per day for 3 days. On day 3, the concentration of plasma bitopertin and the percentage inhibition of ^3^H-glycine uptake were determined 1, 4, 8, or 12 hours after dosing. Schematic was created with BioRender.com (Schmidt, P., 2025; https://BioRender.com/oefao6p). The relationship between the inhibition of glycine uptake and drug concentration is displayed as 12-hour average inhibition and 12-hour average free plasma concentration of bitopertin. (**C**) Female rats were treated by gavage with 0, 0.1, 0.3, 1, and 3 mg/kg bitopertin once per day for 7 days. On days 0 (black) and 7 (red), the concentration of plasma bitopertin and the percentage inhibition of ^3^H-glycine uptake were determined 0.5, 2, 4, 8, or 12 hours after dosing. The relationship between the inhibition of glycine uptake and drug and concentration is displayed as 12-hour average inhibition and 12-hour average free plasma concentration of bitopertin.

To quantitate the extent to which bitopertin can inhibit the uptake of glycine in vivo, we dosed male C57BL/6J mice with 0.3–30 mg/kg bitopertin by oral gavage for 3 days and female Wistar rats with 0.1–3 mg/kg bitopertin by oral gavage for 7 days (Figure 4A). PB contains reticulocytes and RBCs. In rodents, more so than in humans, reticulocytes retain their ability to synthesize heme as demonstrated by the expression of key enzymes in the pathway (Supplemental Figure 2) (40). The plasma concentration of bitopertin and the ability of PB cells to take up ^3^H-glycine were determined ex vivo. The EC_50_ of the effect of bitopertin on glycine uptake was 1.2 nM and 2.0 nM in mice (Figure 4B) and rats (Figure 4C), with a maximal inhibition of 55% and 65%, respectively. In the rat, the effect of bitopertin was not different in the animals treated for either 1 day or 7 days, indicating that there was no tachyphylaxis to the inhibitory effect. Overall, these analyses indicate that bitopertin could inhibit RBC glycine uptake at doses that were readily achieved with oral administration.

### Bitopertin treatment in mouse models of XLPP and EPP

We fed cohorts of C57BL/6N-Alas2^Gln548*/Y^ and C57BL/6J-Fech^m1PAS/m1PAS^ (referred to here as Fech^–/–^) animals, modeling XLPP and EPP, respectively, with chow containing bitopertin for 8 weeks. The phenotypic severity of protoporphyria differs in these mouse models (41). EPP animals have a mild microcytic anemia at baseline, and most develop liver disease. The relative phenotypic severity of EPP mice is likely related to the inheritance of a severe homozygous loss-of-function mutation, resulting in approximately 5% residual FECH activity, which is substantially less residual activity than occurs in most patients who retain 30%–35% of activity. Adult male EPP animals had RBC PPIX levels that were approximately 2–3 times higher than in male XLPP animals (Supplemental Figure 3F and Supplemental Figure 4F). The baseline RBC PPIX level varies with age, peaking at around 4 weeks and stabilizing at around 8 weeks of age in untreated EPP animals (41, 42). Even at older ages, the inter-animal variability in PPIX fluorescence was large, especially in EPP animals, with a coefficient of variation of 30%–40%, reflecting disease heterogeneity even in these genetically identical mice.

**Figure 5:**
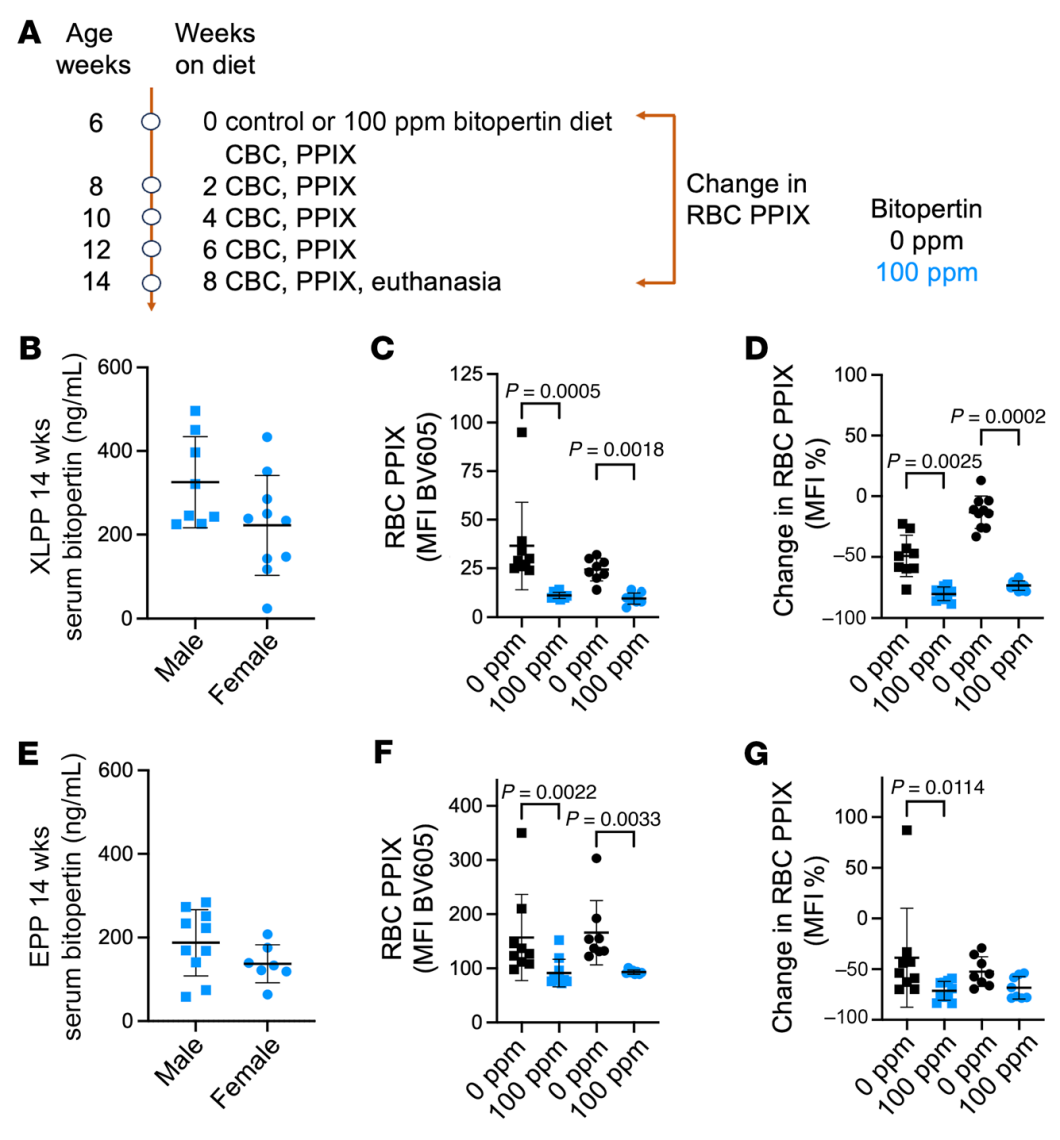
PPIX accumulation and erythroid differentiation in XLPP and EPP mice fed 100 ppm bitopertin for 8 weeks, up to 14 weeks of age. (**A**) Treatment and sampling schema. (**B**) Serum bitopertin concentrations after 8 weeks on 100 ppm bitopertin in male (squares) and female (circles) XLPP animals (14 weeks of age). (**C**) RBC PPIX MFI in XLPP animals. (**D**) Percentage change in RBC PPIX MFI between baseline and 14 weeks of age in XLPP animals. (**E**) Serum bitopertin concentrations after 8 weeks on 100 ppm bitopertin in male (squares) and female (circles) EPP animals. (**F**) RBC PPIX MFI in EPP animals. (**G**) Percentage change in RBC MFI from baseline (0%) until 14 weeks of age in EPP animals. *P* values were calculated using the 2-tailed Mann-Whitney *U* test (**B** and **E**) or the Kruskal-Wallis test with Dunn’s multiple-comparison test (all others). Data are presented as the mean ± SD.

We recorded biweekly changes in RBC parameters and PPIX accumulation, and we assessed erythroid differentiation at the end of the experiment (Figure 5A and Supplemental Figure 3I). The serum concentration of the drug in XLPP animals fed 100 ppm bitopertin was 100–500 ng/mL (Figure 5B), comparable to levels achieved in humans at dose levels currently being evaluated in clinical trials (43). The serum drug concentration in EPP animals was approximately one-third lower than that in XLPP animals (Figure 5E). It is unclear if this difference in systemic exposure to bitopertin was due to the specific protoporphyria allele, to differences in the strain background (i.e., C57BL/6J vs. C57BL/6N), to lean body mass difference between the XLPP and EPP animals, or to other unknown factors, all of which may possibly lead to differences in drug metabolism.

#### Treatment of XLPP mice with 100 ppm bitopertin.

Bitopertin treatment had little to no effect on Hb, but the mean RBC volume (MCV) and other RBC indices changed in a manner consistent with the inhibition of heme synthesis (Supplemental Figure 3, A–E). As anticipated, the MFI of RBC PPIX was significantly reduced: approximately 30% and 40% in treated XLPP male and female animals compared with the animals that received no treatment (Figure 5, C and D). We confirmed these results by quantitative analysis of RBC PPIX using ultra-high-pressure liquid chromatography (UPLC) at necropsy (Supplemental Figure 3, F and G). The distribution of immature erythroid cell populations in bone marrow was not affected by bitopertin treatment in XLPP animals. We observed no significant decrease in PPIX in any immature erythroid cell subpopulations in the bone marrow, despite a trend toward a decrease in PPIX accumulation in more mature cells of the treated animals (Supplemental Figure 3, J–P).

#### Treatment of EPP mice with 100 ppm bitopertin.

The effects on Hb and RBC parameters on EPP animals treated with 100 ppm bitopertin were generally similar to those seen in XLPP animals (Supplemental Figure 4, A–E). However, consistent with the lower drug levels and the more severe phenotype, the observed decrease in RBC PPIX in EPP animals was less than in the XLPP model (Figure 5, F and G, and Supplemental Figure 4, F and G). The distribution of erythroid cell populations in the marrow and spleen (i.e., stress erythropoiesis) was not affected by drug treatment. Similarly, the accumulation of PPIX in erythroid subpopulations was not different (Supplemental Figure 4, I–R).

### Optimized dosing of bitopertin in EPP mice ameliorates RBC PPIX accumulation

#### Treatment of EPP mice with 200 ppm bitopertin.

**Figure 6:**
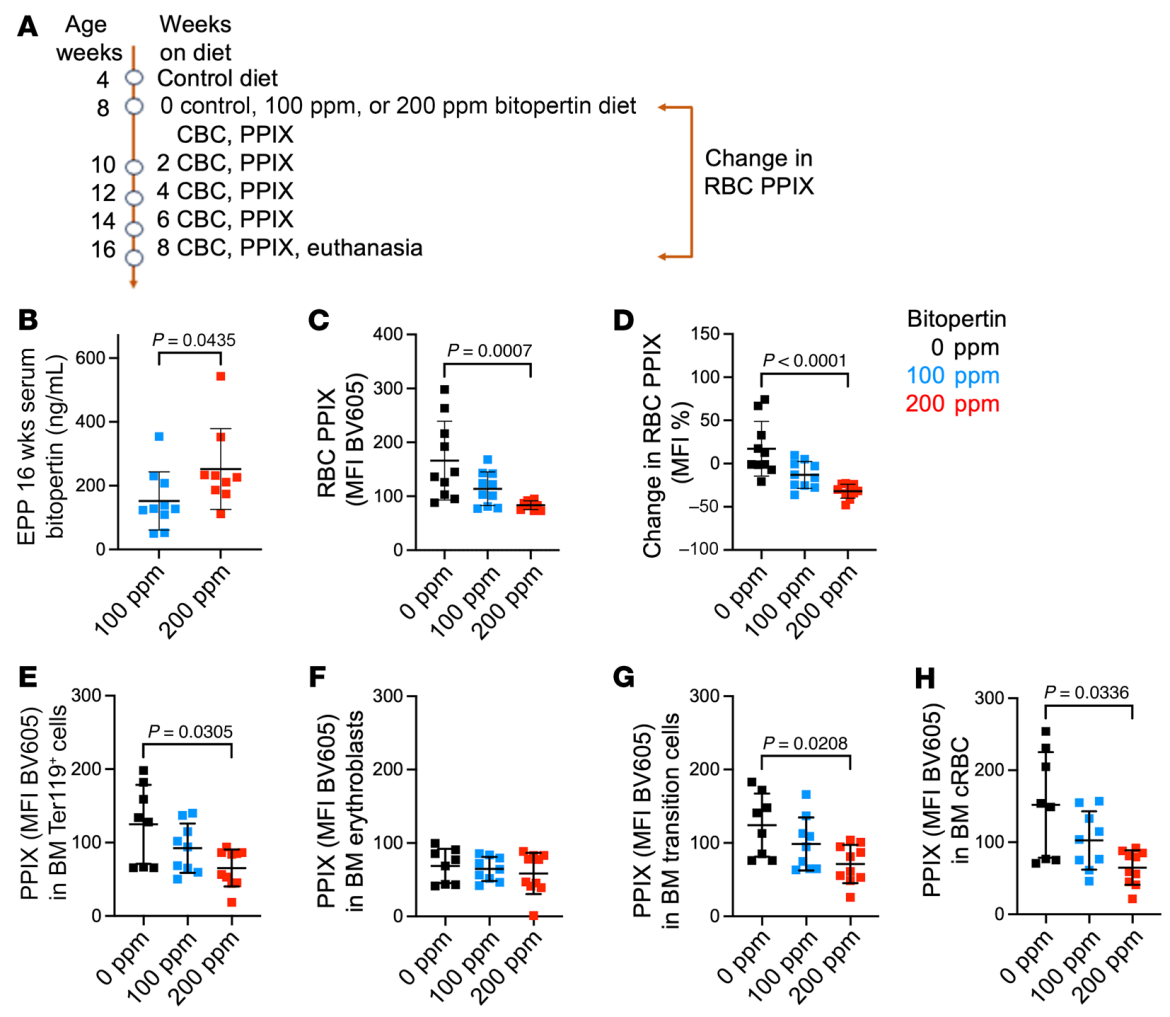
PPIX accumulation and erythroid differentiation in male EPP mice fed 100 or 200 ppm bitopertin for 8 weeks, up to 16 weeks of age. (**A**) Treatment and sampling schema. (**B**) Serum bitopertin concentrations in male EPP animals after 8 weeks on the diet (16 weeks of age). (**C**) RBC PPIX MFI. (**D**) Percentage change in RBC PPIX MFI between baseline and 16 weeks of age. (**E**–**H**) Bone marrow RBC PPIX quantification. PPIX MFI in (**E**) all Ter119^+^ cells, (**F**) erythroblasts, (**G**) transitional erythroblasts/reticulocytes, and (**H**) mature RBC (cRBCs) populations in the bone marrow after 8 weeks. Gating for **E**–**H** is as in Supplemental Figure 3I. *P* values were calculated using a 2-tailed Mann-Whitney *U* test (**B**) or the Kruskal-Wallis test with Dunn’s multiple-comparison test (all others). Data are presented as the mean ± SD.

To reduce the effect of changes in RBC PPIX during development in EPP mice, we performed a follow-up experiment, beginning drug treatment when mice were 8 weeks of age (Figure 6A). We also increased the dose level of bitopertin to 200 ppm to obtain higher drug levels. Feeding EPP male mice chow containing 200 ppm bitopertin resulted in drug levels comparable to those in XLPP males fed 100 ppm bitopertin (Figure 5B and Figure 6B). Whereas RBC indices did not differ between 100 ppm and 200 ppm drug treatment, both diets resulted in a more pronounced hypochromic, microcytic anemia compared with control chow–fed animals (Supplemental Figure 5, A–E). Only the 200 ppm dose significantly reduced RBC PPIX levels (Figure 6, C and D, and Supplemental Figure 5G). Analyses of bone marrow and spleen demonstrated a limited dose-dependent effect on erythropoiesis and stage of maturation, while still reducing erythroid PPIX. The greatest reduction in PPIX MFI was observed in marrow reticulocytes and circulating RBCs in the animals treated with 200 ppm bitopertin (Figure 6, E–H, and Supplemental Figure 5, H–M).

#### Rapid onset of bitopertin effects.

**Figure 7:**
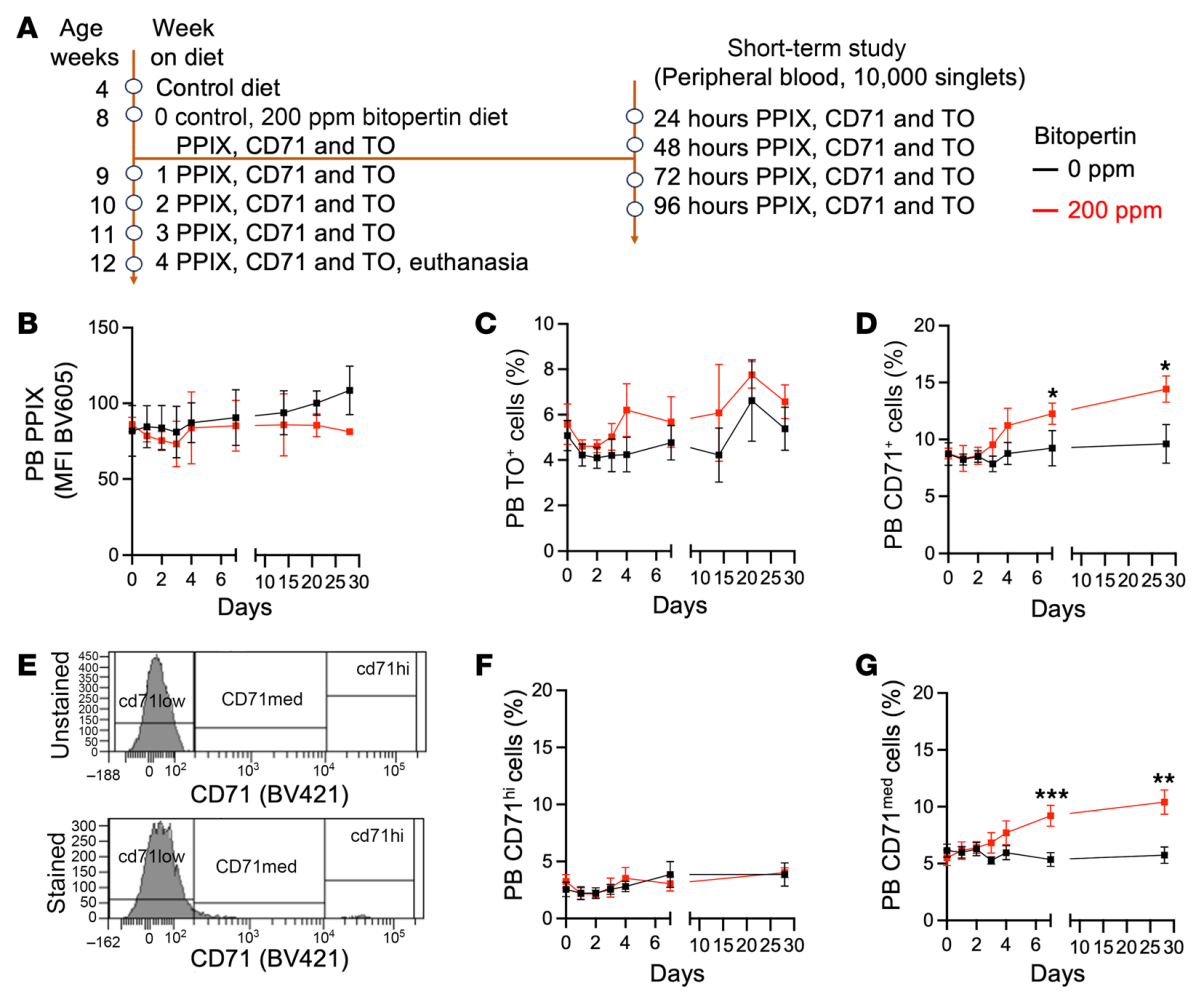
Percentage of reticulocytes in EPP mice fed a 200 ppm bitopertin diet. (**A**) Treatment and sampling schema. Pretreatment and drug treatment are the same as the 200 ppm arm described in Figure 6A, however, blood sampling and preparation were modified to examine the reticulocyte and RBC response to bitopertin treatment after hours and days. All animals were EPP. (**B**) PPIX MFI in circulating PB cells at the indicated time points following the introduction of a 200 ppm bitopertin diet (red) or maintenance on the control diet (black). (**C**) The reticulocyte fraction was measured by TO^+^ staining versus the time after introduction of the bitopertin diet. (**D**) The reticulocyte fraction was measured by CD71 (transferrin receptor) expression (CD71^+^ = CD71^hi^ + CD71^med^) versus the time after introduction of the bitopertin diet. (**E**) Flow cytometric analytical strategy to determine populations in CD71-stained cells. (**F**) Fraction of CD71^hi^ reticulocytes versus the time after introduction of the bitopertin diet. (**G**) Fraction of CD71^med^ cells versus the time after introduction of the bitopertin diet. *P < 0.05, **P <0.01, and ***P < 0.001, by unpaired, 2-tailed Welch’s *t* test with Holm-Šídák’s multiple-comparison test. Data are presented as the mean ± SD.

**Figure 8:**
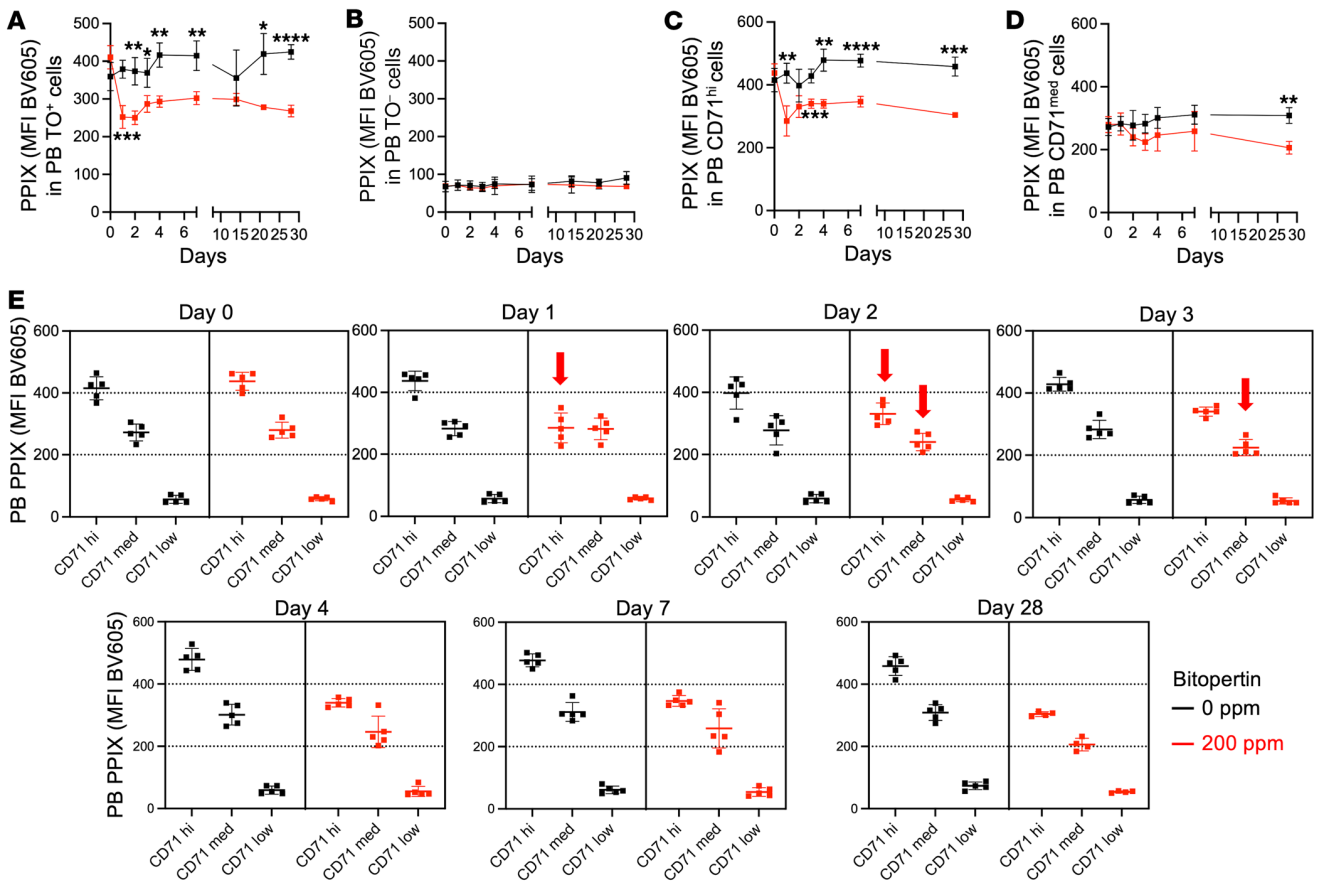
PPIX accumulation in reticulocytes and erythrocytes of EPP mice fed a 200 ppm bitopertin diet. (**A**) PPIX in TO^+^ (reticulocytes) PB versus the time after introduction of the bitopertin diet. (**B**) PPIX in TO^–^ (erythrocytes) PB versus the time after introduction of the bitopertin diet. (**C**) PPIX in CD71^hi^ (young reticulocytes) PB versus time after introduction of bitopertin diet. (**D**) PPIX in CD71^med^ (young erythrocytes) PB versus the time after introduction of the bitopertin diet. (**E**) Comparison of PPIX MFI in CD71^hi/med/lo^ PB populations versus time in EPP animals fed the control or 200 ppm bitopertin diet. Data are replotted from **C** and **D** along with CD71^lo^ (older erythrocytes) population. Note the rapid decrease in PPIX on day 1 in the CD71^hi^ population that could then be observed over the next 2 days in the CD71^med^ population, as indicated by the red arrows. *P < 0.05, **P <0.01, ***P < 0.001, and ****P < 0.0001, by unpaired, 2-tailed Welch’s *t* test with Holm-Šídák’s multiple-comparison test. Data are presented as the mean ± SD.

Because the maximum effect on the RBC PPIX MFI occurred much more rapidly (<14 days) after drug introduction than the expected RBC lifespan (40–50 days), we evaluated whether PPIX was differentially distributed in reticulocytes compared with older RBCs in EPP mice (Figure 7A). In untreated animals, PPIX MFI in reticulocytes (i.e., erythroid cells that were thiazole orange^+^ [TO^+^], CD71^hi^, or CD71^med^) was higher than PPIX MFI in RBCs (i.e., erythroid cells that were unstained, TO^–^, or CD71^lo^) (Figure 7, B–G, and Figure 8, A–E), demonstrating that, in mice, PPIX accumulated in reticulocytes and was lost during RBC aging. The percentage of TO^+^ cells was unaffected by treatment (Figure 7C), whereas the percentage of CD71^+^ cells increased (Figure 7D). Analysis of intermediate cell populations showed that the proportion of CD71^med^ cells was the one increasing (Figure 7, F and G). Tfr1/CD71 expression is iron-dependent, and, consequently, after bitopertin exposure, protein levels may remain elevated in later stages of reticulocyte maturation to facilitate further incorporation of iron into PPIX. Bitopertin treatment preferentially reduced PPIX levels in the reticulocytes, and the effect could be seen after just 1 day of drug exposure (Figure 8, A, C, and D). Reticulocytes eventually established a new steady-state PPIX level that was one-half to two-thirds as high as that in untreated cells but still approximately 3 times higher than in TO^–^ or CD71^lo^ RBCs. This result demonstrates that the rapidity of the response to bitopertin was a consequence of its effects on reticulocytes, which disproportionately contributed to PPIX levels in the RBCs.

### Effect of bitopertin treatment on EPP liver disease

**Figure 9:**
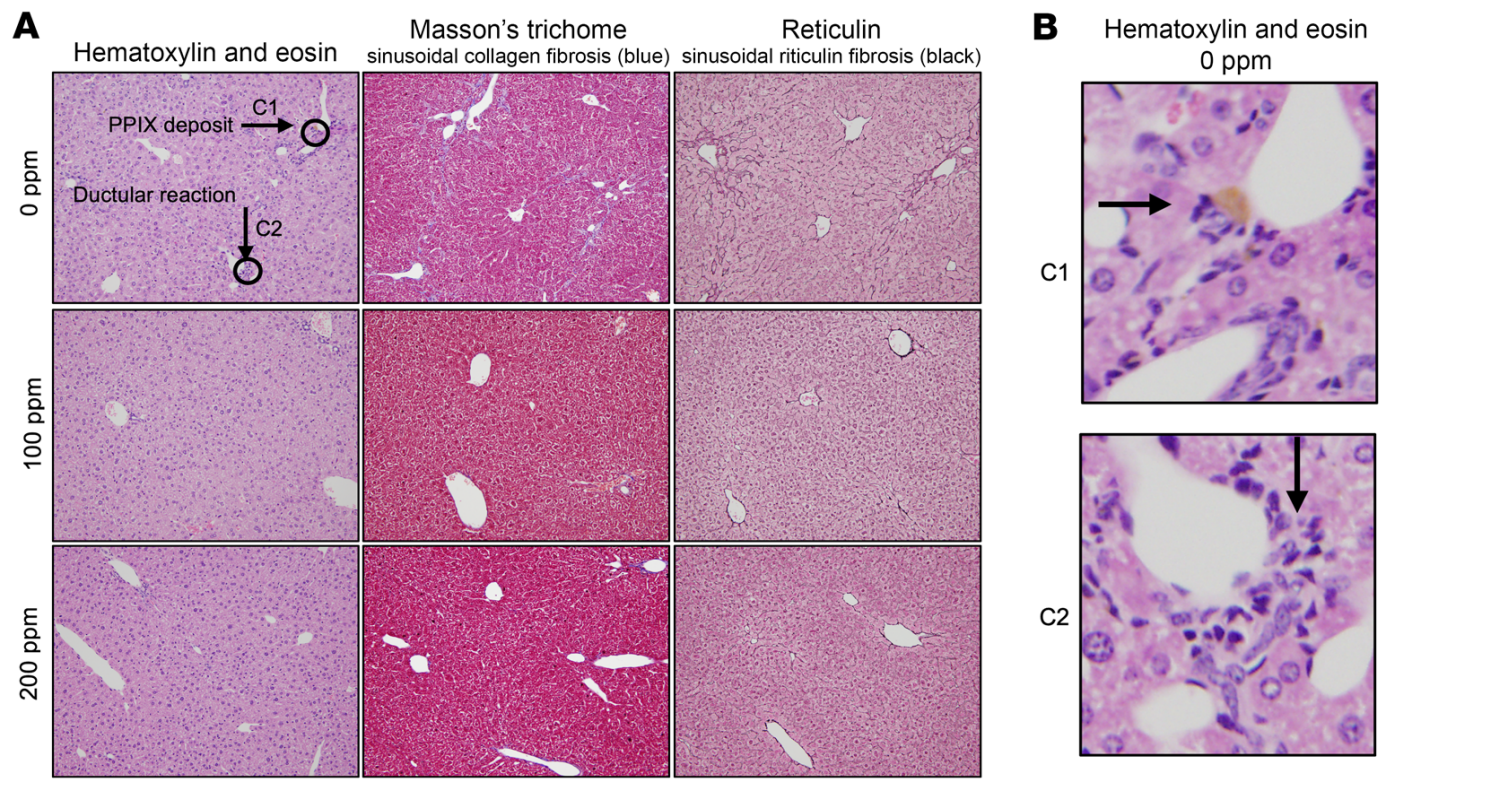
Liver histopathology in response to bitopertin treatment in EPP mice. (**A**) Histopathology images of representative EPP animals fed 0 (untreated) or 100 ppm or 200 ppm bitopertin for 8 weeks. Original magnification, ×10. Histology was performed and reviewed for all animals. Note the increase in blue Masson’s trichrome staining and black reticulin staining in the untreated animal and less reticulin staining and absent trichrome staining in the 100 ppm animal. Both staining patterns are absent in the 200 ppm example. (**B**) Enlarged view of PPIX deposits (arrow, top) and ductular reaction (arrow, bottom) in control-fed animals. Original magnification, ×10 (A, top left). C1, control enlargement 1 (as defined in **A**, top left); C2, control enlargement 2 (as defined in **A**, top left).

**Figure 10:**
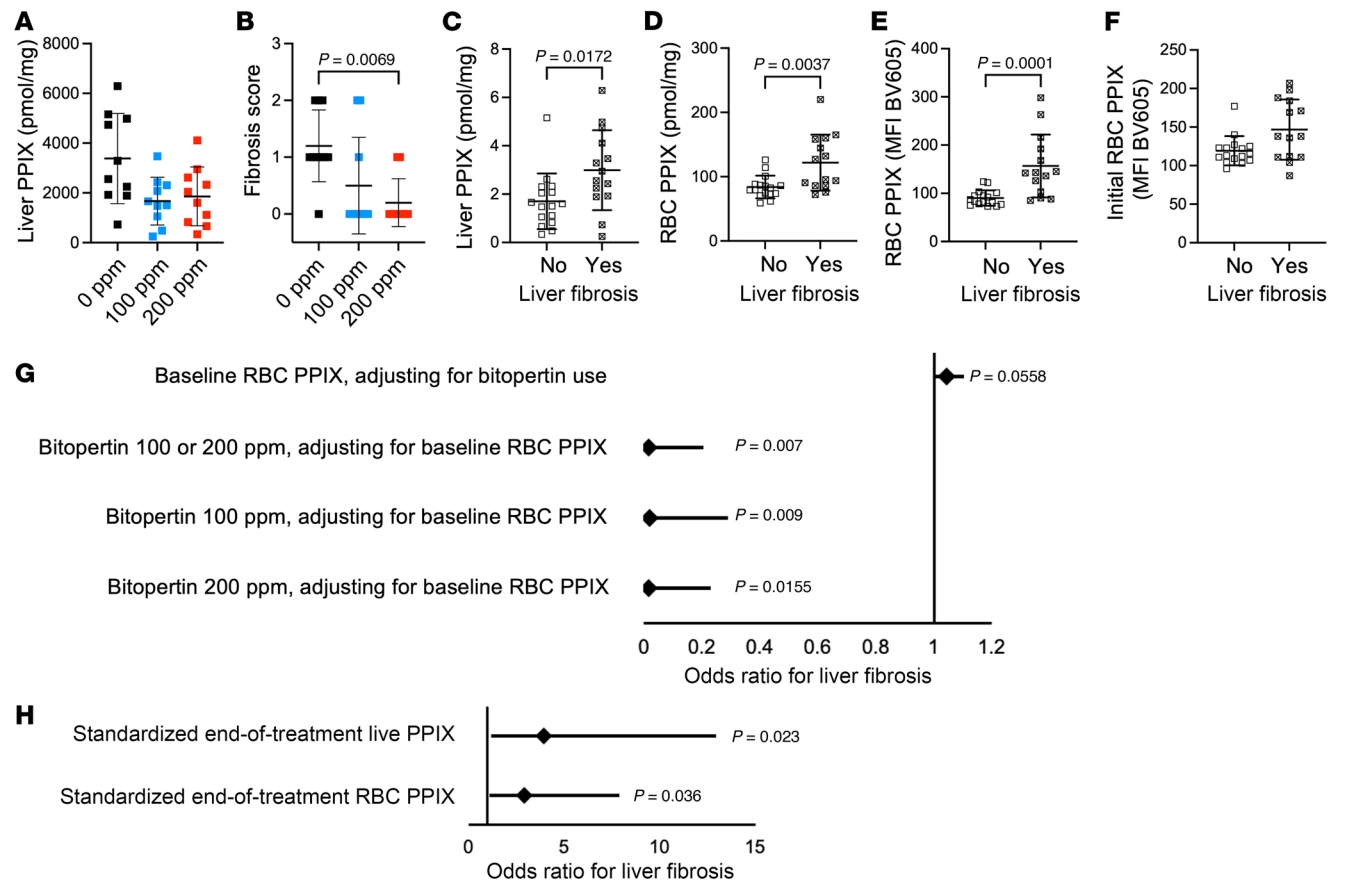
Liver response to bitopertin treatment in EPP mice. (**A**) Quantification of liver PPIX levels after 8 weeks on bitopertin. (**B**) Histological liver fibrosis score after 8 weeks on bitopertin. Liver fibrosis at age 16 weeks was associated with (**C**) liver PPIX accumulation, (**D**) RBC PPIX levels, and (**E**) RBC PPIX MFI, as well as (**F**) trends toward increased initial RBC PPIX, irrespective of bitopertin therapy. (**G**) Multivariate logistic regression analysis to determine the OR for liver fibrosis associated with baseline RBC PPIX, adjusting for bitopertin treatment, and bitopertin treatment, adjusting for baseline RBC PPIX. (**H**) Logistic regression analysis to determine the OR for liver fibrosis that was associated with a standardized 1-unit increase in liver PPIX and RBC PPIX. Both liver and RBC PPIX levels were standardized to a mean of 0 and a SD of 1. *P* values were calculated using the Kruskal-Wallis test with Dunn’s multiple-comparison test (**A** and **B**) or a 2-tailed Mann-Whitney *U* test (**C**–**F**). Data are presented as the mean ± SD.

At baseline, XLPP animals accumulate only a limited amount of PPIX in the liver, rarely developing liver disease (41); we observed no histological evidence of liver disease in these animals (data not shown). In contrast, EPP animals have a high incidence of liver disease, similar to the protoporphyric hepatopathy described in patients (11), including fibrosis and bile ductular reaction at an early age (42). After 8 weeks of treatment, we found that bitopertin treatment reduced mean liver PPIX levels in EPP mice, although this trend was not statistically significant. Nonetheless, histopathological evaluation of the livers, scored on a categorical scale of 0–4, (Supplemental Table 3 and Figure 9, A and B, and Figure 10A), demonstrated that bitopertin, particularly at the 200 ppm dose, reduced the extent of histological liver fibrosis (Figure 10B). Importantly, none of the mice on the 200 ppm diet developed a fibrosis score of 2 or higher. Furthermore, the presence or absence of fibrosis was significantly associated with both liver and RBC PPIX levels at 8 weeks, irrespective of whether the animal had been treated with the drug (Figure 10, C–E). There was a similar trend toward a presence of liver fibrosis in animals with higher initial RBC PPIX levels (*P* = 0.0682, Figure 10F). Liver aminotransferases (aspartate transferase [AST] and alanine aminotransferase [ALT]) or bilirubin levels were not significantly affected by drug treatment (Supplemental Figure 6). However, no animal treated with 200 ppm bitopertin had evidence of hyperbilirubinemia, whereas 30% and 20% of those receiving 0 ppm or 100 ppm drug, respectively, did (Supplemental Figure 6B).

Given the potential effect of the initial RBC PPIX levels on liver fibrosis, we performed multivariable logistic regression analyses to examine (a) the interaction between RBC PPIX levels prior to drug therapy and (b) the effect of drug treatment on the presence or absence of liver fibrosis at the end of the study. Normalizing to the baseline PPIX MFI, we found that treatment with (a) 100 ppm, (b) 200 ppm, or (c) 100 or 200 ppm bitopertin for 8 weeks strongly reduced the odds ratio (OR) for the occurrence of liver fibrosis (OR: 0.0196, 95% CI: 0.0004–0.2905; OR: 0.0161, 95% CI: 0.0004–0.2294; OR: 0.0175, 95% CI: 0.0004–0.2045; respectively; Figure 10G). The area under the receiver operator curve, which considered the treatment dose, and the baseline MFI yielded an 89% ability to predict the presence or absence of fibrosis (Supplemental Figure 6E). Furthermore, liver and RBC PPIX levels at the end of treatment were both associated with an increased OR of fibrosis (OR: 2.90, 95% CI: 1.21–9.53; OR: 3.95, 95% CI: 1.45–16.79, respectively; Figure 10H).

Although much of the liver damage occurs before the animals are 8 weeks of age (42), the murine EPP liver disease in this model progressed with age, including the evolution of hepatic tumors (44), which are extremely rare in patients with protoporphyria (11). Consequently, we evaluated the effects of long-term 200 ppm bitopertin treatment for 32 weeks in a cohort of adult male and female EPP animals (40-week study, see Supplemental Figure 7A). We found that the positive effect of bitopertin in lowering PPIX levels in RBCs was maintained long term, with only a mild decrease in Hb observed in treated males (Supplemental Figure 7, B–D). Although liver PPIX accumulation was not altered in the 40-week study, several histological and biochemical metrics of liver disease improved in drug-treated animals (Supplemental Figure 7, E–K).

**Figure 11:**
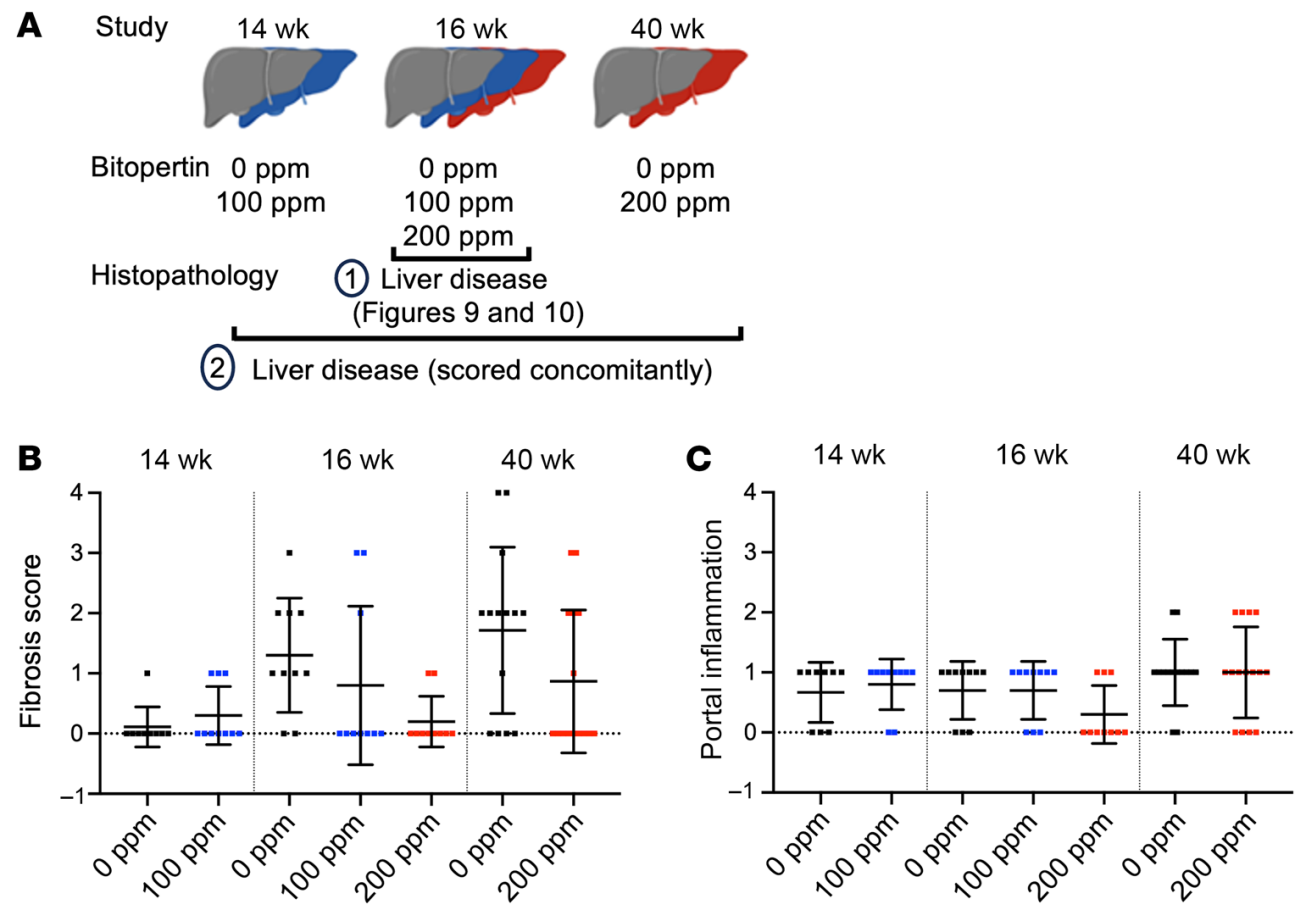
Fibrosis and portal inflammation evolution in male EPP animals enrolled in 14-week, 16-week, and 40-week studies following bitopertin treatment. (**A**) Graphical representation of the concomitant analysis of histopathology parameters (scoring system in Supplemental Table 3). Illustration was created with BioRender (Ducamp, S., 2025; https://BioRender.com/wvh26qu). (**B**) Fibrosis score and (**C**) portal inflammation. Data are presented as the mean ± SD.

Histopathological evaluation was conducted concurrently for all 3 cohorts of EPP animals (14, 16, and 40-week studies), with the 16-week cohort being re-scored (Figure 11A). In untreated male animals, fibrosis and portal inflammation progressed in severity with increasing age, whereas the incidence of ductular reaction did not (Figure 11, B and C, and Table 1). As expected, untreated EPP livers had more significant abnormalities in the 40-week study. A notable histological feature was a hepatocarcinoma (HCC) and dysplastic hepatocellular nodules found in 1 and 3 male animals, respectively, in the untreated group (Supplemental Figure 7, M and N), reflecting the development of more severe cholestatic features (45). Such tumors were not observed in the treated group, but the number of animals was insufficient to conclude a drug benefit. Treatment with 200 ppm bitopertin significantly reduced the incidence of ductular reaction (Table 1) and the severity of fibrosis (Figure 11B and Table 2; *P* = 0.0023) in the livers of the male group. In contrast, the portal inflammation (Figure 11C and Table 2) was unchanged, consistent with the fact that PPIX still accumulates in the liver of treated animals. Detailed reports of the histological evaluations (scoring of PPIX and iron deposition in Kuppfer cells, bile plugs, and lobular inflammation as well as data for females) are provided in the Supporting Data Values file. Altogether, these results suggest that the histopathological features of liver disease associated with EPP disease are ameliorated upon bitopertin treatment. Drugs reducing the production of PPIX, such as bitopertin, could modify the most severe consequence of PPIX accumulation in the liver.

## Discussion

Here, we describe the use of bitopertin, an orally bioavailable small-molecule inhibitor of GLYT1, the RBC membrane glycine transporter, to achieve a substantial reduction in RBC PPIX accumulation. GLYT1 is a compelling pharmacological target due to its tissue specificity, which is largely limited to the brain and erythroid cells, the existence of a second glycine transporter (GLYT2) in other tissues, and the unique requirement of erythroid cells to acquire glycine for heme synthesis, which necessitates 8 glycines per heme molecule. We established the EC_50_ of bitopertin in a model of EPP erythroblasts and demonstrate that concentrations of bitopertin in range of the EC_50_ efficiently reduced PPIX accumulation in erythroblasts derived from 3 unrelated patients with EPP. Additionally, we demonstrate that bitopertin significantly reduced erythroid PPIX accumulation in vivo in the EPP and XLPP mouse models. Interestingly, in mice, PPIX accumulated predominantly in reticulocytes. Our data show that the bitopertin effect was the most potent in this erythroid subpopulation. This is consistent with the fact that, in rodents, in contrast to humans, heme synthesis peaks in late erythroblasts and reticulocytes (Supplemental Figure 2).

While it is expected that bitopertin decreases the pool of glycine directly available for heme production, it is also possible that diminished glycine uptake affects other metabolites important for heme synthesis and erythropoiesis. For example, glycine is also required for the synthesis of glutathione (GSH), which protects RBCs from oxidative stress (46). In addition, work by several groups (47–52) has demonstrated that loss of the mitochondrial GSH importer SLC25A39 diminishes heme synthesis and the activity and stability of iron sulfur clusters, resulting in alterations in iron metabolism and impaired RBC production. Consequently, it is conceivable that diminished GSH production may play a role in the bitopertin-induced reduction in erythrocyte PPIX burden.

Although we did not test whether bitopertin affects phototoxicity in this study, whole-blood PPIX levels have been associated with symptom severity and sun tolerance in patients (53). In a small cohort, extracorporeal light inactivation of PPIX also decreased the degree of light sensitivity (54). Thus, even a partial reduction in RBC PPIX levels could significantly improve photosensitivity and the adverse effects of light avoidance on quality of life (55). Another preclinical study recently demonstrated that the administration of an analog of bitopertin resulted in diminished phototoxicity in the BALB/C EPP mouse model (56). Preliminary data from an open-label pilot study of bitopertin in patients with EPP have shown reductions in whole-blood PPIX levels of a magnitude comparable (40%–60%) to that reported in this study (57). These preliminary clinical data were further confirmed in a randomized, double-blind, placebo-controlled study, in which bitopertin (60 mg) treatment resulted in a significant and sustained decrease in whole-blood PPIX levels relative to placebo (40%, *P* < 0.001) (58).

We also provide evidence that bitopertin can modify the liver phenotype of EPP mice. EPP animals treated with 200 ppm bitopertin for both 8 and 32 weeks showed evidence of less severe liver disease. Prevention of liver pathology is difficult to achieve in these mice, probably because damage begins early in life (42). In adult EPP animals, only partial recovery of liver function and histological alterations have been reported even after bone marrow transplantation, which fully corrects the excess RBC PPIX production (59). Liver disease is life-threatening in 2%–5% of patients with EPP, and its treatment is a significant unmet need (60–62). Given the mouse data presented here, bitopertin might also ameliorate this phenotype in humans. Histological evidence of ductular reaction, portal inflammation, and fibrosis, commonly observed in liver biopsies from patients with EPP (11), was present in most of the untreated EPP mice studied. As in humans, only a subset of EPP mice developed very severe liver disease. Consequently, it is not practical to determine the efficacy of bitopertin in reducing the most acute, life-threatening complication of EPP in this model system. Nonetheless, we conclude that bitopertin can reduce both ductular reaction and fibrosis in this model of EPP, providing evidence that it modifies the underlying pathological processes leading to liver disease in EPP mice.

The primary measured side effect in this preclinical study was reduced Hb. This is not unexpected, as this was observed in clinical studies of bitopertin in schizophrenia and is an expected consequence of a reduction in heme synthesis. Initial data from the open-label study of bitopertin in EPP (57, 63) showed that stable levels of Hb and no anemia adverse events. It is also noteworthy that in our HSC model of EPP, we could measure large reductions of PPIX accumulation in response to bitopertin without an appreciable effect on erythropoiesis. The differential effect on anemia in the mouse may be due to the extreme severity of the *Fech*^m1PAS^ allele (~5% residual activity) compared with the loss of enzymatic activity in most patients with EPP (~25–35% residual activity), the dose and schedule of the drug, or other factors. In total, however, the data reported in this study strongly support the continued evaluation of bitopertin as a potential therapy for the treatment of EPP and XLPP. A confirmatory clinical trial (APOLLO) evaluating bitopertin in patients with EPP or XLPP is planned for initiation by mid-2025.

## Methods

### Sex as a biological variable.

This study examined male and female animals, and sex-dimorphic effects are reported.

### K562 EPP model.

CRISPR/Cas9 was used to generate a null variant (indel leading to a premature stop codon) in *trans* of the c.315-48T>C variant (Supplemental Table 1) in K562 cells (ATCC).

### HSC FECH shRNA model.

Human umbilical cord blood stem cells (Human Cord Blood CD34 Stem/Progenitor Cells, Hemacare) were transduced with lentiviruses encoding a control or *FECH*-specific shRNA (Supplemental Table 2), differentiated into erythroblasts, and analyzed on day 16 for erythroid differentiation, *FECH* mRNA and protein expression, PPIX fluorescence by flow cytometry, and ALA, PPIX, and heme production by liquid chromatography/mass spectrometry.

### EPP human erythroid differentiation.

PB was obtained from 3 different patients with EPP. CD34^+^ progenitors (HSCs) were purified using magnetic anti-CD34 microbeads (Miltenyi Biotec) and differentiated into erythroblasts using an established 2-stage protocol with minor modifications (64). Bitopertin in DMSO was added to the media at a final concentration of 0, 10, or 50 nM. On alternate days, PPIX was quantified by flow cytometry, and morphologic erythroid maturation was assessed.

### Glycine uptake in bitopertin-treated mouse and rat erythrocytes.

Female Wistar rats or male C57BL/6J mice were treated with bitopertin via oral gavage for 7 or 3 days, respectively. On the indicated days, the plasma concentration of bitopertin was determined, and the ability of PB erythrocytes to take up ^3^H-glycine was determined in vitro at different time points after administration of the dose. The relationship between the inhibition of glycine uptake and drug concentration was calculated as the 12-hour average inhibition versus the 12-hour average free plasma concentration of bitopertin.

### Animals.

EPP *Fech^m1Pas/m1Pas^* mice (65) were maintained on the C57BL/6J background. An XLPP allele, Alas2^Gln548*^, corresponding to a common XLPP pathogenic variant in patients (3), was created by CRISPR/Cas9-mediated transgenesis of C57BL/6N embryos (41). EPP and XLPP animals were maintained in the barrier facility at Boston Children’s Hospital. Pregnant dams (Fech^–/+^ or Alas2^Gln548*/+^) were fed the Prolab RMH 3000 diet.

### Bitopertin treatment of EPP/XLPP mice.

Mice were subjected to 3 different treatment regimens. All pups were weaned onto Prolab RMH 3000 and switched to a semisynthetic diet when indicated. (a) Initial study (14 weeks): Male and female EPP and XLPP animals were switched to a diet supplemented with 0 or 100 ppm bitopertin (control diet Envigo TD.120277 and TD.200425, respectively) at 6 weeks of age and maintained on the control or drug diet for 8 weeks. (b) Dose-escalation study (16 weeks): EPP male mice were placed on the control diet at 4 weeks of age and maintained on that diet until 8 weeks of age. At that time, the cohort was divided into a control diet arm and 2 treatment arms: 100 or 200 ppm (Envigo TD.210209) bitopertin for a total of 8 weeks. (c) Short-term study: Male and female EPP animals were fed according to scheme 2 above, and at 8 weeks of age, animals of each were sex allocated to remain on the control diet or transferred to the 200 ppm bitopertin diet for up to 4 weeks. (d) Long-term study (40 weeks): EPP male mice were fed according to scheme 2 above, and at 8 weeks of age, animals were allocated to remain on the control diet or transferred to the 200 ppm bitopertin diet for up to 32 weeks of treatment (40 weeks of age).

Blood was sampled retro-orbitally for complete blood counts and PPIX quantification by flow cytometry at baseline and periodically thereafter as indicated. At necropsy, blood and liver tissue were collected for serum analyses, histopathology, and quantification of heme and PPIX concentrations by flow cytometry and UPLC.

### Flow cytometry.

Relative RBC PPIX levels were determined using a violet excitation laser and emission fluorescence at 610/20 nm (i.e., BV605) on a BD Celesta flow cytometer and expressed as MFI. PPIX was quantified in fresh samples with this method requiring as little as 1 μL PB. Erythroid differentiation was evaluated in the marrow and the spleen as previously described (66). For the reticulocyte study, tail-vein blood was stained with TO or CD71-BV421. PPIX MFI was determined in unstained, TO^+^, TO^–^, CD71^hi^, CD71^med^, and CD71^lo^ cells. Data were analyzed with Diva or FlowJo software.

### Quantitative heme and PPIX quantification.

Total RBC/liver heme and PPIX analyses were performed by UPLC. Measurements were performed on frozen RBC pellets or tissue, retrospectively, to limit batch-to-batch variation, and normalized by total protein with this method.

### Serum biochemical analyses.

Total bilirubin, AST, and ALT analyses were performed on a Roche Cobas c501 chemistry analyzer. For the 40-week study, these analyses, along with alkaline phosphatase (ALP) and γ-glutamyl transferase (GGT), were performed with colorimetric kits (Abcam AB241029 and MilliporeSigma MAK126-1KT, MAK467-1KT, MAK052-1KT, and MAK447-1KT) following the manufacturers’ protocols.

### Histopathology.

Conventionally prepared microscopic slides were evaluated by a fellowship-trained liver pathologist in a blinded fashion and scored for fibrosis, macrophage and hepatocyte porphyrin accumulation, iron staining, portal and lobular inflammation, and bile plugs on a scale of 0–3 or 0–4 (Supplemental Table 3). Ductular proliferation and reaction was noted. In the 40-week study, all discreet hepatic nodules observed grossly were also sampled for histopathological assessment. The 14-week study was scored twice, first independently and then alongside the livers from the other studies. No statistical differences were found between the 2 sets of scores, despite the presence of more damaged livers in the 40-week study (data not shown). Both independent scores have been kept (Figures 9–11 and Tables 1 and 2).

### Statistics.

Data were analyzed in GraphPad Prism 10.1.1 (GraphPad Software) or Rstudio software, version 3.5.3. Differences among treatment groups were determined by an unpaired, 2-tailed *t* test with Welch’s correction or a Mann-Whitney *U* test for comparisons between 2 groups. The Kruskal-Wallis test with Dunn’s multiple-comparison test was used for analysis among 3 or more groups. *P* values in Figures 7 and 8 were calculated using an unpaired, 2-tailed *t* test with Welch’s correction and a Holm-Šídák multiple-comparison test. The ORs for fibrosis were determined by logistic regression. For the final set of analyses (fibrosis score, portal inflammation, and ductular reaction), the statistical tests were conducted only for male animals. Two types of tests were performed on the basis of the variable nature. For analyses of the fibrosis score and portal inflammation, a 2-factor ANOVA model without interaction was applied. For analysis of ductular reactions, 2 separate multiple logistic regression models were required, as not all doses were evaluated in all studies (see Supplemental Methods). Data are presented as the mean ± SD, except for the ORs, which are displayed with 95% CIs. A *P* value of less than 0.05 was considered significant.

### Study approval.

Patients with EPP were enrolled in a human subjects research protocol approved by Boston Children’s Hospital, and written informed consent was received for each participant. EPP and XLPP animals were maintained in the barrier facility at Boston Children’s Hospital under protocols approved by the institution. Control female Wistar rats and male C57BL/6J mice were maintained under a protocol approved by Disc Medicine.

### Data availability.

Relevant information about the data will be made available directly from the corresponding authors. See Supplemental Methods for complete details. Values for all data points in graphs are reported in the Supporting Data Values file.

## Author contributions

SD designed experiments, created the XLPP model, developed and performed PPIX and erythroid differentiation flow cytometry experiments, performed EPP HSC-erythroblast differentiation, analyzed and interpreted data, and wrote the manuscript. MW designed experiments and provided oversight of the studies, generated in vitro results using the K562-EPP cell line and human WT erythroblasts, provided oversight concerning the glycine uptake assay using mouse and rat erythroid cells, analyzed and interpreted data, and wrote the manuscript. JP analyzed the mouse histopathology results. DRC performed mouse phenotyping. YX designed experiments, developed and performed the RBC glycine uptake assays, analyzed and interpreted data, and wrote the manuscript. VH generated the K562-EPP cell line and evaluated the effect of bitopertin in K562-EPP cells and in human WT erythroblasts. MMH recruited patients. KKD analyzed a portion of the liver data (14W study) and recruited patients. RKL recruited patients. MDF designed experiments and wrote the manuscript. BM designed experiments and wrote the manuscript. PJS designed experiments, provided project oversight, performed mouse experiments, analyzed and interpreted data, and wrote the manuscript.

## Supplementary Material

Supplemental data

Unedited blot and gel images

Supporting data values

## Figures and Tables

**Table 2:**
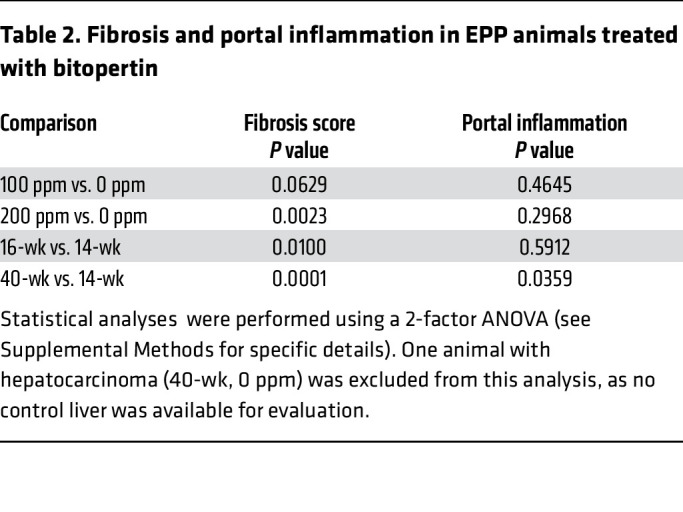
Fibrosis and portal inflammation in EPP animals treated with bitopertin

**Table 1:**
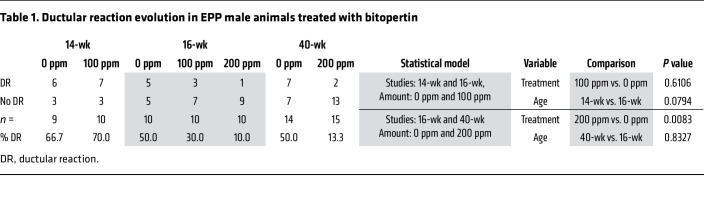
Ductular reaction evolution in EPP male animals treated with bitopertin

## Version 1 07/15/2025

In-Press Preview

## Version 2 09/16/2025

Electronic publication
