# Supplementary material for: The GLYT1 inhibitor bitopertin mitigates erythroid PPIX production and liver disease in erythroid protoporphyria
Source: J Clin Invest. 2025 Jul 15;135(18):e181875. doi: 10.1172/JCI181875 (PMC12435834; doi:10.1172/JCI181875)
Supplement: Supplemental data [file jci-135-181875-s045.pdf]

SUPPLEMENTAL FILES

SUPPLEMENTAL METHODS

K562 EPP model. A *FECH* null allele was created in K562 erythroleukemia cells (ATCC) with sgRNA-1 using CRISPR/Cas9 technology (Supplemental Table 1). sgRNA-1 was used to target exon 3 of the FECH genetic locus to generate the single cell clone 10, which carries a 1 bp deletion of FECH gene at the KO allele, resulting in the generation of a premature stop codon (p.Thr81fs8*). The c.315-48T>C variant was subsequently targeted into clone 10 using sgRNA-2 as the single-stranded template of reparation.

Embryonic human hematopoietic stem cell FECH shRNA model. Human umbilical cord blood stem cells (Human Cord Blood CD34 Stem/Progenitor Cells, Hemacare) were transduced with lentiviruses encoding a control or *FECH*-specific shRNA at a multiplicity of infection of 1:25 (Supplemental Table 2). Lentivirally-infected cells were selected with puromycin on days 4-7 after infection and treated with defined concentrations of bitopertin in erythroid differentiation medium beginning on day 8, and analyzed on day 16 for erythroid differentiation, *FECH* mRNA and protein expression (FECH, 14466-1-AP, Proteintech; GAPDH, D16H11 XP® Rabbit mAb, Cell Signaling Technology), mf-PPIX fluorescence by flow cytometry, and 5-aminolevulinic acid (5-ALA), PPIX and heme production by liquid chromatography/mass spectrometry (LC/MS).

Adult human EPP erythroid differentiation. 50 ml of peripheral blood were obtained from 3 independent EPP patients enrolled in a human subjects research protocol approved by Boston Children's Hospital. All three patients were compound heterozygous for the c.315-48T>C and a predicted null *FECH* allele (p.L322fs*14, p.K379* and S281Rfs*55). Blood products were manipulated with limited light to decrease PPIX toxicity. $CD34^+$ progenitors (hematopoietic stem cells, HSCs) were purified using magnetic anti-CD34 microbeads (Miltenyi Biotec), yielding a purity >95%. HSCs were differentiated using a 2-stage protocol (1) (with minor modifications).

Briefly, $CD34^+$ cells were cultured for 7 days in Iscove Dulbecco's Modified Eagle Medium (IMDM) containing 15% BIT 9500 serum substitute (StemCell Technologies), 100 ng/mL Stem Cell Factor (SCF), 10 ng/mL interleukin (IL)-6 and 10 ng/mL IL-3 (IIS media). On day *7*, Epo (10 U/mL) was added to the media (EIS media). 10% fetal bovine serum was added on day *8* to promote EPP erythroid cells survival. CD36 magnetic sorting was performed to enrich the culture in erythroid progenitors using magnetic microbeads (CD36 FA6.152 from Beckman Coulter and anti-mouse IgG1 MicroBeads from Miltenyi Biotec) when enough cells were obtained for each patient. DMSO or bitopertin (resuspended in DMSO at final concentration of 10 or 50 nM) was added to EIS media at D02, D06, and D08 for patients EPP001, EPP002, and EPP003, respectively, prior to the cells starting to accumulate significant PPIX. For patients EPP002 and EPP003, DMSO-treated cells were split on day 14, and 10 nM bitopertin was added to one-half of the culture to determine the effect of the drug on cells already accumulating mf-PPIX. Each EIS culture was counted and diluted to 0.4-0.8 million cells every other day. Beginning on EIS day 8, and every other day thereafter, PPIX was quantified by flow cytometry using $1x10^5$ cells. Erythroid maturation was determined using May-Grünwald-Giemsa (MGG) stained cytocentrifuged cells at each time point.

Glycine uptake in bitopertin-treated mouse and rat erythrocytes. Female Wistar rats were treated with bitopertin at 0, 0.1, 0.3, 1, and 3 mg/kg once per day via oral gavage for 7 days. Blood was collected at 0.5, 2, 4, 8, and 12 hours (hr) post dosing on day 0 and day 7 from 2 rats per group. Similarly, male C57BL/6J mice were treated with bitopertin at 0, 0.3, 1, 3, 10, and 30 mg/kg once per day for three days via oral gavage. Blood was collected at 1, 4, 8, and 12 hr post dosing on day 3. The plasma concentration of bitopertin was determined using LC-MS/MS. The ability of peripheral blood erythrocytes to take up ^{3}H-glycine was determined *in vitro*. RBCs from treated animals were harvested from whole blood by centrifugation and were incubated with 600 nM ^{3}H-glycine at room temperature for 30 min and ^{3}H-glycine uptake was measured by liquid scintillation.

The 12 hr average glycine uptake inhibition was calculated as the % inhibition AUC/12 hr. The 12 hr average bitopertin concentration was calculated by the AUC of the free plasma bitopertin concentration/12 hr. The relationship of percentage inhibition to the concentration of bitopertin was analyzed using GraphPad Prism and the EC_{50} was calculated using nonlinear fit with four parameters.

Animals. *Fech$^{m1Pas/m1Pas}$* (abbreviated Fech$^{-/-}$) mice were maintained on the C57BL/6J background.(2) An XLPP allele, Alas2^{Gln548*}, corresponding to a common XLPP mutation in patients, was created by CRISPR/Cas9-mediated transgenesis of C57BL/6N embryos (3). Animals were kept in the barrier facility at Boston Children's Hospital under approved protocols. Pregnant dams (Fech$^{-/+}$ or Alas2$^{Gln548*/+}$) and pups up to 4 or 6 weeks of age were fed Prolab RMH 3000 diet.

Bitopertin treatment of EPP/XLPP mice. Protoporphyric mice were subjected to three different treatment regimens: 1) 14W - initial study: Fech$^{-/-}$ male and females and Alas2$^{Gln548*/Y}$ males and Alas2$^{Q548/+}$ females weaned on the Prolab RMH 3000 diet were switched to a semisynthetic control diet (Envigo TD.120277) or the control diet supplemented with 100 ppm bitopertin (Envigo TD.200425) at age 6 weeks and maintained on drug for 8 weeks. 2) 16W - drug escalation study: Fech$^{-/-}$ male mice were weaned onto the Prolab RMH 3000 diet and switched onto the control diet at 4 weeks of age and maintained on that diet until 8 weeks of age at which time the cohort was divided into a control diet and two treatment arms: 100 ppm or 200 ppm (Envigo TD.210209) bitopertin for a total of 8 weeks. 3) short term study: To determine the short-term effect of bitopertin on reticulocyte mf-PPIX, male and female Fech$^{-/-}$ animals were fed according to scheme 2 above. At age 8 weeks, animals of each sex were allocated to remain on the control diet or transferred to the 200 ppm bitopertin diet. 4) 40W - long term study: To determine the long-term effect of bitopertin, Fech$^{-/-}$ male mice were weaned onto the Prolab RMH 3000 diet and switched onto the control diet at 4 weeks of age and maintained on that diet until 8 weeks of age. At this time, the

cohort was divided into a control diet or maintained on drug (200 ppm (Envigo TD.210209)) for a total of 32 weeks.

In each case, peripheral blood was sampled retro-orbitally for complete blood counts (CBCs) and mf-PPIX quantification at baseline and as indicated in Figures 5A, 6A, 7A and Supplemental 7A thereafter, until the termination of the experiment. CBCs were performed at the Center for Virology and Vaccine Research Flow Cytometry Core, Beth Israel Deaconess Medical Center using a Bayer Advia 120 instrument. At necropsy, blood and tissue were collected for serum analyses, RBC and liver quantitative heme and PPIX determination, and histopathology.

Flow cytometry. Qualitative mf-PPIX levels were determined by flow cytometry using the violet excitation laser and recording fluorescence at 610/20 nm (*i.e.*, BV605) on a BD Celesta flow cytometer and expressed as mean fluorescence intensity (MFI). Erythroid differentiation was evaluated in the marrow and the spleen by flow cytometry as previously described (4) using ter119-PE (BioLegend 116208) and CD44-APC (BD biosciences 559250). For the short-term study, 10 μl tail bleeds were obtained on days 0, 1, 2, 3, 4, 7, 14, and 21 and reticulocytes in diluted blood were stained with 1/500 thiazole orange (TO, diluted at 1mg/ml in methanol) or CD71-BV421 (Biolegend 113813) for 10 minutes shaking in the dark at room temperature. PPIX accumulation was determined in unstained, TO^+, $CD71^{high}$, $CD71^{med}$ and $CD71^{low}$ cells. Data were preliminarily acquired with the BD DIVA software (version 8.0.1.1) and reanalyzed using FlowJo (version 10).

Quantitative heme and PPIX quantification. Total heme and mf-PPIX analyses were performed at the Iron and Heme Core facility at the University of Utah, supported by a grant from the NIH National Institute of Diabetes and Digestive and Kidney Diseases, grant number U54DK110858, employing a Waters Acquity ultra-high pressure liquid chromatography (UPLC) system equipped

with a reverse-phase C_{18} column and tandem high sensitivity photodiode array and fluorescence detectors.

Serum biochemical analyses. Total bilirubin, aspartate aminotransferase (AST), and alanine aminotransferase (ALT) analysis were performed on serum diluted 1:1 in phosphate buffered saline using a Roche Cobas c501 chemistry analyzer. For the 40w study, these analyses, along with alkaline phosphatase (ALP) and gamma glutamyl transferase (GGT), were performed with colorimetric kits (abcam AB241029 and Sigma-Aldrich MAK126-1KT, MAK467-1KT, MAK052-1KT, MAK447-1KT) following manufacturers' protocols.

Histopathology. Livers were fixed in 10% buffered formalin for 24 hours and embedded in paraffin. Deparaffinized sections of tissue were stained with H&E, PAS, iron, reticulin, or Masson's trichrome stain in the Boston Children's Hospital, Department of Pathology Histology Laboratory. Images were acquired using a 10X/0.30 objective lens on a BX51 microscope equipped with a DP71 Digital Camera employing Olympus CellSens Entry version 1.12. Conventionally prepared microscopic slides were evaluated by a fellowship-trained liver pathologist (JP) in a blinded fashion and scored for fibrosis, macrophage and hepatocyte porphyrin accumulation, iron staining, portal and lobular inflammation and bile plugs on a scale of 0-3 or 0-4 (Supplemental Table 3). Ductular proliferation/reaction were noted. Gross pathological abnormalities were observed on untreated animals in the 40W study and livers were assessed for hepatocarcinoma. Initially, only the livers of the 16W study were scored. All the livers were scored simultaneously after the completion of the 40W study, including the 16W study livers that were reassessed. No significant difference was observed between the two assessments.

Statistics. Data were analyzed in Prism v.10.1.1 or Rstudio software version 3.5.3. Differences among treatment groups were determined by an unpaired t-test with Welch's correction or Mann-Whitney test for comparison between 2 groups. The Kruskal-Wallis test with Dunn's multiple

comparisons test was used for analysis among 3 or more groups. *P*-values in Figures 7 and 8 were calculated using an unpaired t-test with Welch correction and Holm Sidak's multiple comparison test. The odds ratios for fibrosis were determined by logistic regression. For the final set of analyses (fibrosis score, portal inflammation, and ductular reaction), the statistical tests were conducted only in male animals. The 16W study contained no female mice. The p-value for an effect of sex in the 40W study was 0.0940 in a two-factor ANOVA model including "sex" and "treatment" as predictors of fibrosis score, indicating a potential relationship between sex and disease progression. Two types of tests were conducted based on the variable nature. Analyses of fibrosis score and portal inflammation used a two-factor ANOVA model without interaction with "treatment" (reference = 0ppm) and "study" (reference = 14W) as predictors. For the analysis of ductular reaction, two separate multiple logistic regression models were required as not all doses were evaluated in all studies. Each model included independent variables of "treatment" (reference = 0ppm), "study" (reference = 16W), and "treatment x study" interaction. The first model included data from 14W and 16W studies for the 0ppm and 100ppm doses. The second model included data from the 16W and 40W studies for the 0ppm and 200ppm doses. The 16W study was chosen as the common reference level for both models due to the inclusion of all three dose groups within this study. Data are presented as mean ± standard deviation (SD), except for the odds ratios, which are displayed with 95% confidence intervals. A *P*-value of less than 0.05 was considered significant.

Supplemental Table 1. Oligonucleotides used in construction and validation of K562-EPP cells

Name	Sequence
**sgRNA-1**	ATGGGAGGCCCTGAAACTCT
**FECH-KO-GT_F**	GTATCTGTGTGACGGCAGTG
**FECH-KO-GT_R**	GCAAAGGGCTCAAGGAGAAT
**sgRNA-2**	TTGAGTAGAAAACATTTCTC
**Single stranded donor DNA**	GGGGGTTCGGCGTTTGGCGATGAATGGTGCCAGCTTACTAAA<br>TCATTTAACATACAGGTAAGTGGATTTTATTCCAGCTTAGCAGC<br>CTGAGAAATGTTTTCTACTCAATAAAAAAGAAAAAAAGCAA
**FECH-IVS-GT_F**	GTTTCTCTGCATGGGTGTTG
**FECH-IVS-GT_R**	GACAATTCATCCAGCAGCTTC
**FECH-CDS-F**	ATGCGTTCACTCGGCGCAAAC
**FECH-CDS-R**	TCACAGCTGCTGGCTGGTGAA
**FECH-RTPCR-F**	GCCCTGAAACTCTTGGAGATG
**FECH-RTPCR-R**	CTGCGGTACTGCTCTTGAATC
**FECH-qPCR-F2**	CATGGAGGTGGAAGTCAGGTG
**FECH-qPCR-R2**	TCAGGGCCTCCCATGTTTAG
**GAPDH-qPCR-F**	GAAGGTGAAGGTCGGAGTC
**GAPDH-qPCR-R**	GAAGATGGTGATGGGATTTC

Supplemental Table 2. shRNAs

Name	Sequence
**shCTRL-GFP**	CCGGCGTGATCTTCACCGACAAGATCTCGAGATCTTGTCGGTGAAGATCACGTTTTT
**shFECH-1**	CCGGGCTTTGCAGATCATATTCTAACTCGAGTTAGAATATGATCTGCAAAGCTTTTT

Supplemental Table 3. Fibrosis and portal inflammation scale.

Fibrosis	Portal Inflammation
0:None	0:None
1:Portal Fibrosis	1:Mild
2:Periportal Fibrosis	2:Moderate
3:Bridging Fibrosis	3:Severe
4:Cirrhosis	

LITERATURE CITED

1. Gautier EF, Ducamp S, Leduc M, Salnot V, Guillonneau F, Dussiot M, et al. Comprehensive Proteomic Analysis of Human Erythropoiesis. *Cell Rep.* 2016;16(5):1470-84.
2. Boulechfar S, Lamoril J, Montagutelli X, Guenet JL, Deybach JC, Nordmann Y, et al. Ferrochelatase structural mutant (Fechm1Pas) in the house mouse. *Genomics.* 1993;16(3):645-8.
3. Ducamp S, Sendamarai AK, Campagna DR, Chin DWL, Fujiwara Y, Schmidt PJ, et al. Murine models of erythroid 5ALA synthesis disorders and their conditional synthetic lethal dependency on pyridoxine. *Blood.* 2024;144(13):1418-32.
4. Chen K, Liu J, Heck S, Chasis JA, An X, and Mohandas N. Resolving the distinct stages in erythroid differentiation based on dynamic changes in membrane protein expression during erythropoiesis. *Proc Natl Acad Sci U S A.* 2009;106(41):17413-8.

Supplemental Figure 1

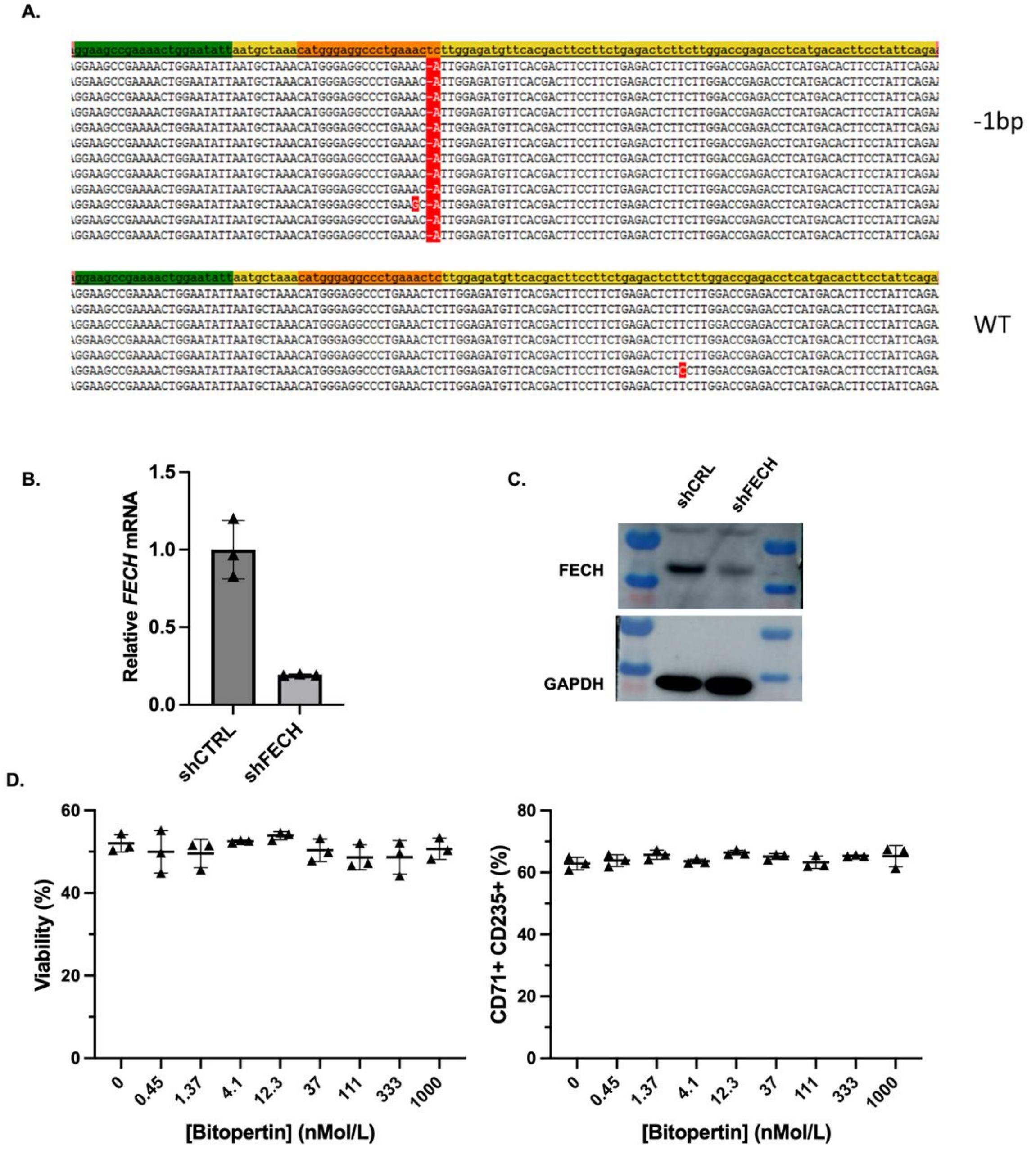

Supplemental Figure S1. Supporting data for Figures 1 and 2. (A) TA clone sequencing for single cell clone 10. **(B)** *FECH* mRNA and **(C)** FECH protein are reduced by FECH shRNA in human umbilical vein hematopoietic stem cells (HSCs). **(D)** Bitopertin treatment does not alter cell viability (left) or erythroid commitment (right) in shRNA-treated human umbilical vein HSCs induced to differentiate in vitro.

Supplemental Figure 2

A.

Gautier et al Blood Advance 2020
Mouse primary erythroblasts
↓
Ex vivo differentiation
↓
Proteomic data
Data extraction (copy number per cell)
TFRC (TFR1): iron importer
ALAS2: 1st enzyme of heme synthesis
(sole source of 5-ALA)
FECH: last enzyme of heme synthesis
GLYT1: glycine importer (Bitopertin target)

Erythroid composition at key time points
T36h 30% Polychromatic erythroblasts
+ 25% Orthochromatic erythroblasts
+ 15% reticulocytes
T48h 50% reticulocytes
T60h >75% reticulocytes

B.

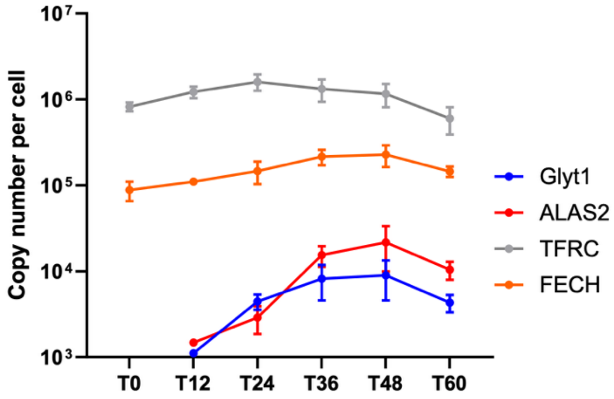

Supplemental Figure S2. (A) Data extraction from proteomic analysis of differentiated mouse primary erythroblasts published online. **(B)** Expression of key enzymes of the murine heme pathway over time.

Supplemental Figure 3 (XLPP 14w)

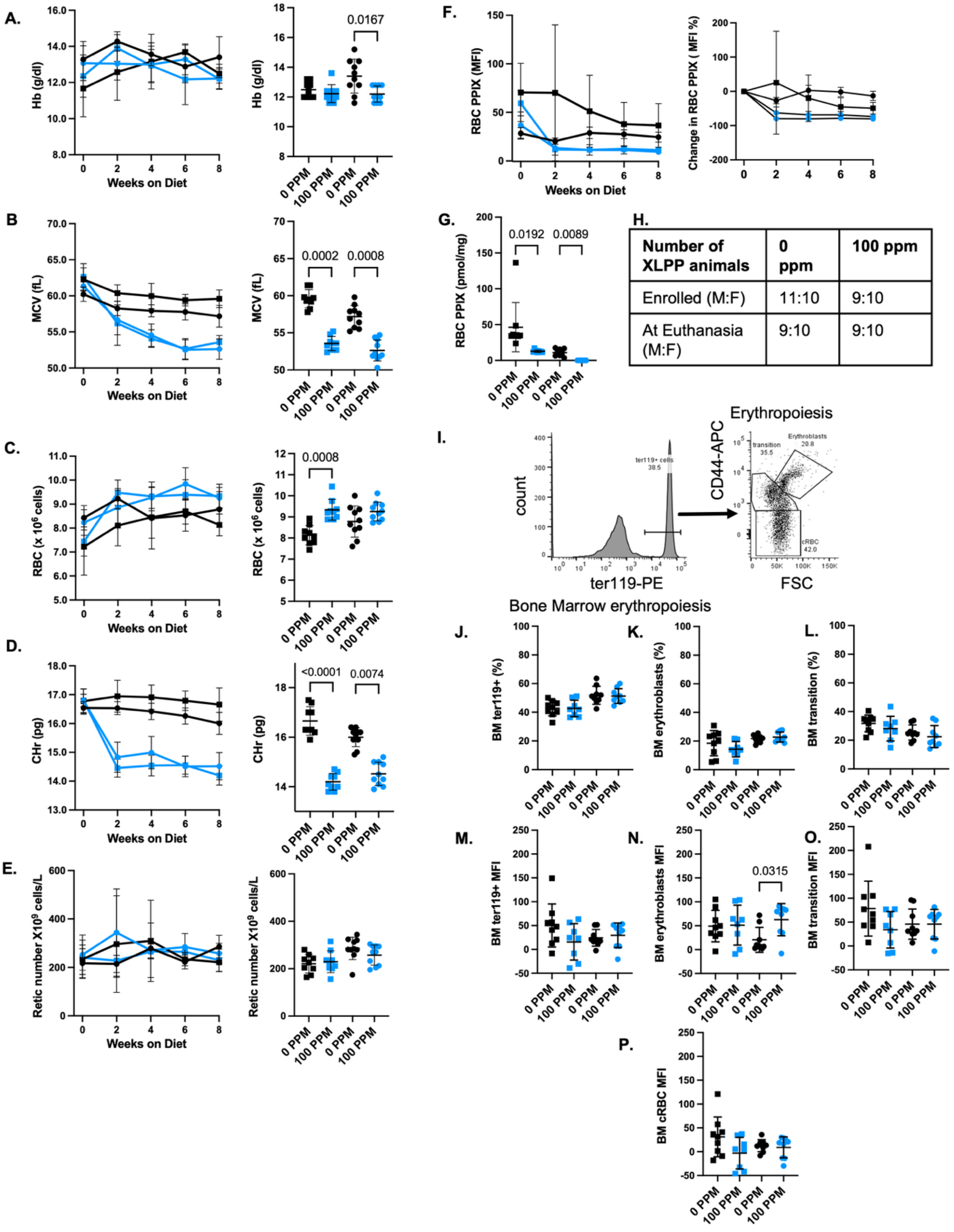

Number of XLPP animals	0 ppm	100 ppm
Enrolled (M:F)	11:10	9:10
At Euthanasia (M:F)	9:10	9:10

Supplemental Figure S3. Supporting data for Figures 5B-D. XLPP (Alas2^{Q548*}) male (squares) and female (circles) animals were fed a control diet (black) or 100 ppm bitopertin diet (blue) for 8 weeks beginning at age 6 weeks. **(A)** Hemoglobin (Hb) over the treatment time course (left) and after 8 weeks on treatment (right). **(B)** Red blood cell mean cell volume (MCV) over the treatment time course (left) and after 8 weeks on treatment (right). **(C)** Red blood cell number (RBC) over the treatment time course (left) and after 8 weeks on treatment (right). **(D)** Cellular hemoglobin of the reticulocyte (CHr) over the treatment time course (left) and after 8 weeks on treatment (right). **(E)** Reticulocyte (Retic) number over the treatment time course (left) and after 8 weeks on treatment (right). **(F)** Absolute (left) and change in RBC mf-PPIX MFI over time (right). **(G)** Quantitative RBC PPIX at takedown. **(H)** Two male animals in the control arm died of unknown causes during the study. **(I)** Flow cytometric analytical strategy for erythropoiesis (Transition: late erythroblasts and reticulocytes, cRBCs: circulating RBCs). **(J-P)** Bone marrow erythroid populations and PPIX MFI in the different subpopulation are unaltered after 8-weeks of bitopertin treatment. *P*-values were calculated using a Kruskal-Wallis test with Dunn’s multiple comparisons test.

Supplemental Figure 4 (EPP 14w)

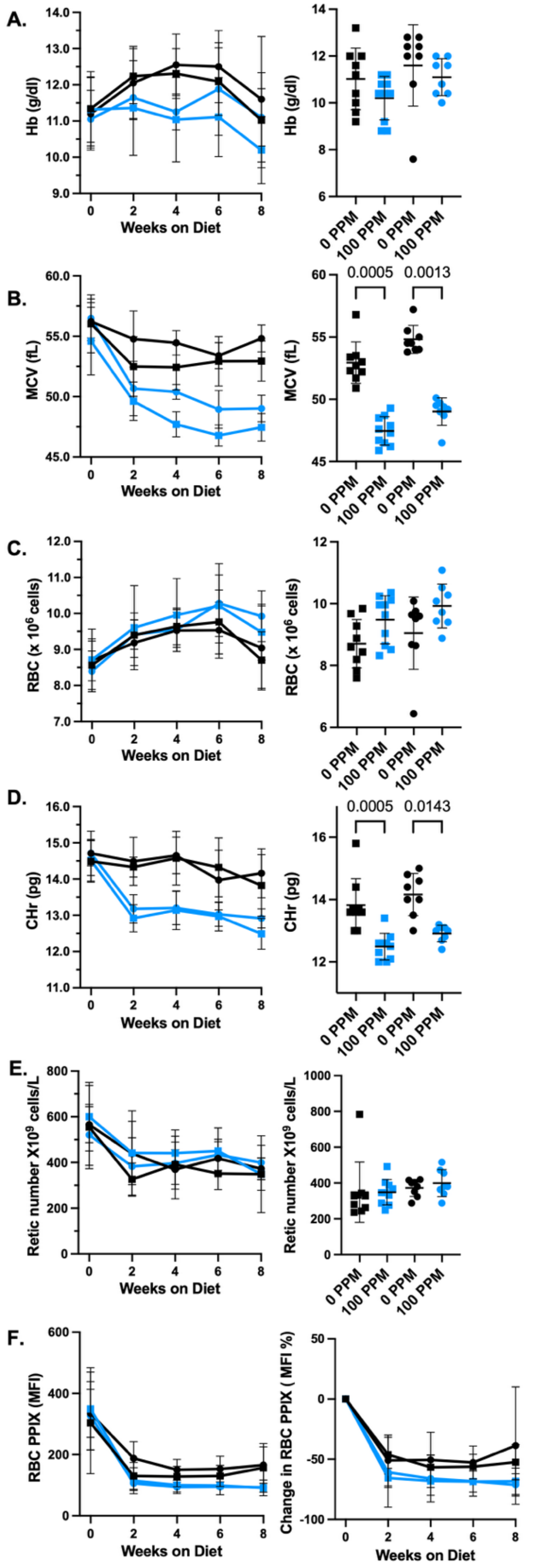

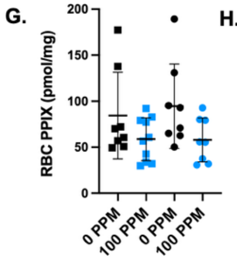

H.

Number of EPP animals	0 ppm	100 ppm
Enrolled (M:F)	10:9	10:8
At Euthanasia (M:F)	9:8	10:8

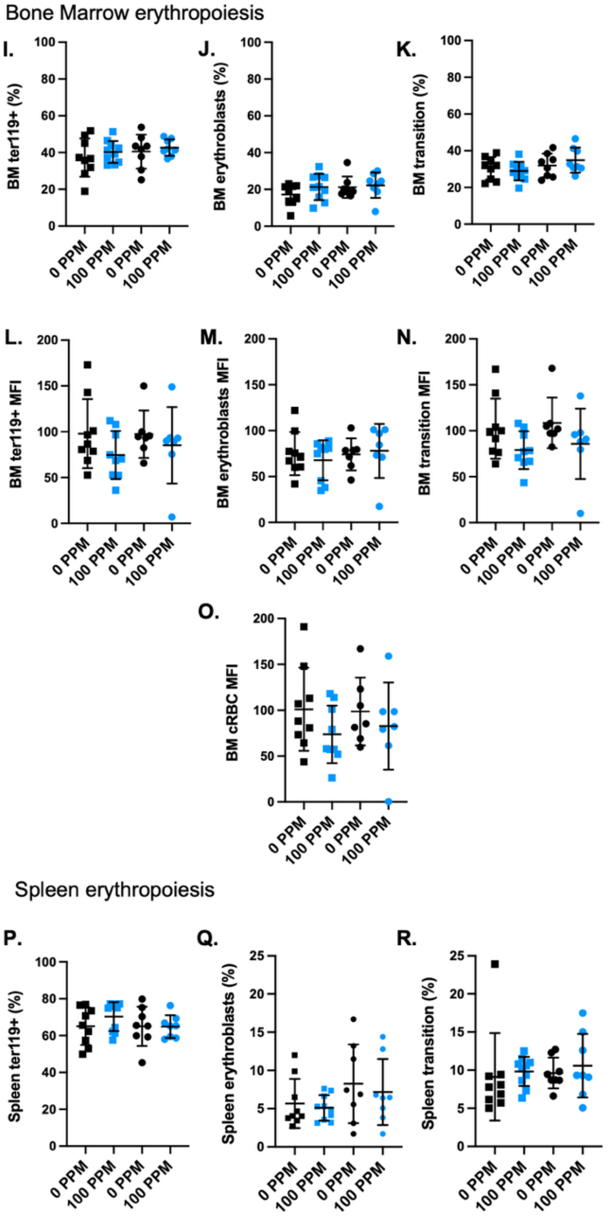

Supplemental Figure S4. Supporting data for Figure 5E-G. EPP (Fech$^{-/-}$) male (squares) and female (circles) animals were fed a control diet (black) or 100 ppm bitopertin diet (blue) for 8 weeks beginning at age 6 weeks. **(A)** Hemoglobin (Hb) over the treatment time course (left) and after 8 weeks on treatment (right). **(B)** Red blood cell mean cell volume (MCV) over the treatment time course (left) and after 8 weeks on treatment (right). **(C)** Red blood cell number (RBC) over the treatment time course (left) and after 8 weeks on treatment (right). **(D)** Cellular hemoglobin of the reticulocyte (CHr) over the treatment time course (left) and after 8 weeks on treatment (right). **(E)** Reticulocyte (Retic) number over the treatment time course (left) and after 8 weeks on treatment (right). **(F)** Absolute (left) and change in RBC mf-PPIX MFI over time (right). **(G)** Quantitative RBC PPIX at takedown. **(H)** One male and one female animal in the control arm were found dead by veterinary staff of unknown causes during three-times weekly health checks at 8- and 6-weeks of age, respectively. **(I-O)** Bone marrow erythropoiesis and mf-PPIX MFI in each subpopulation (ter119+, erythroblasts, transitional erythroblasts/reticulocytes and circulating RBC or cRBC). Gating strategy is depicted in Supplemental Figure S3I. **(P-R)** splenic erythroid populations are unaltered after 8-weeks of bitopertin treatment. *P*-values were calculated using a Kruskal-Wallis test with Dunn’s multiple comparisons test.

Supplemental Figure 5 (EPP 16w)

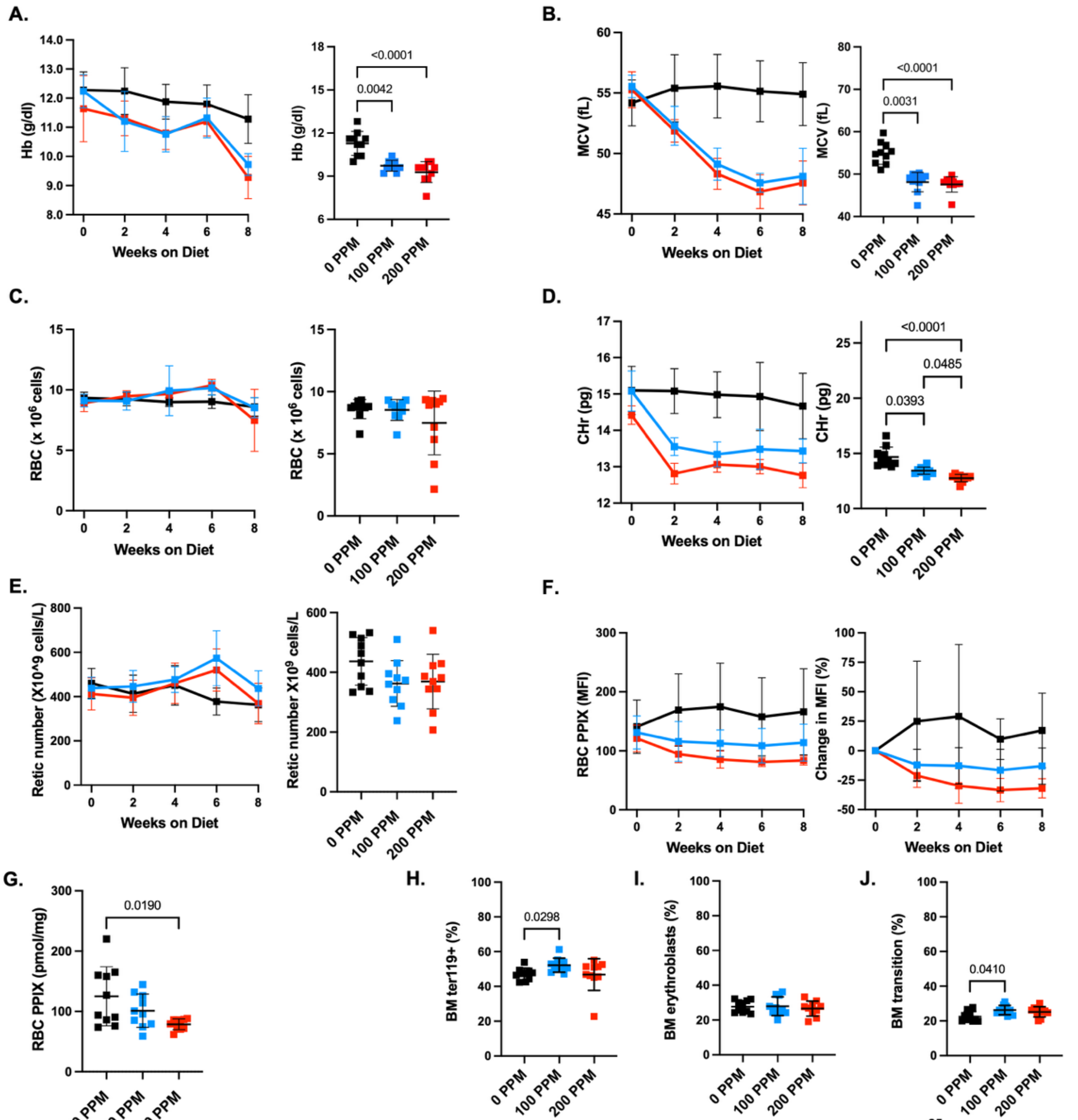

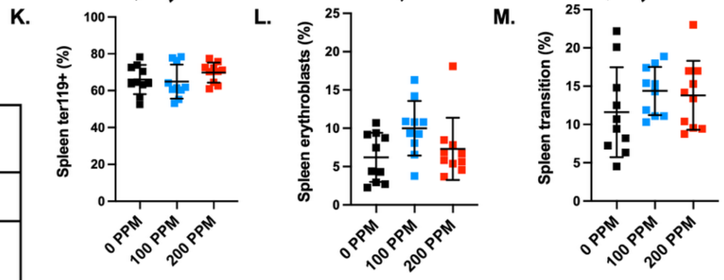

N.

Number of EPP animals	0 ppm	100 ppm	200 ppm
Enrolled (M)	10	10	11
At Euthanasia (M)	10	10	10

Supplemental Figure S5. Supporting data for Figures 6B-H. EPP (Fech$^{-/-}$) male animals were fed a control diet (black), 100 ppm bitopertin (blue), or 200 ppm bitopertin for 8 weeks beginning at age 8 weeks. **(A)** Hemoglobin (Hb) over the treatment time course (left) and after 8 weeks on treatment (right). **(B)** Red blood cell mean cell volume (MCV) over the treatment time course (left) and after 8 weeks on treatment (right). **(C)** Red blood cell number (RBC) over the treatment time course (left) and after 8 weeks on treatment (right). **(D)** Cellular hemoglobin of the reticulocyte (CHr) over the treatment time course (left) and after 8 weeks on treatment (right). **(E)** Reticulocyte (Retic) number over the treatment time course (left) and after 8 weeks on treatment (right). **(F)** Absolute (left) and change in RBC mf-PPIX MFI over time (right). **(G)** Quantitative RBC PPIX at takedown. **(H-J)** Bone marrow and **(K-M)** splenic erythroid populations are unaltered after 8-weeks of bitopertin treatment. **(N)** One male in the 200 ppm arm was euthanized by veterinary staff due to labored breathing and diminished mobility at 10 weeks of age. *P*-values were calculated using a Kruskal-Wallis test with Dunn's multiple comparisons test.

Supplemental Figure 6 (EPP 16w)

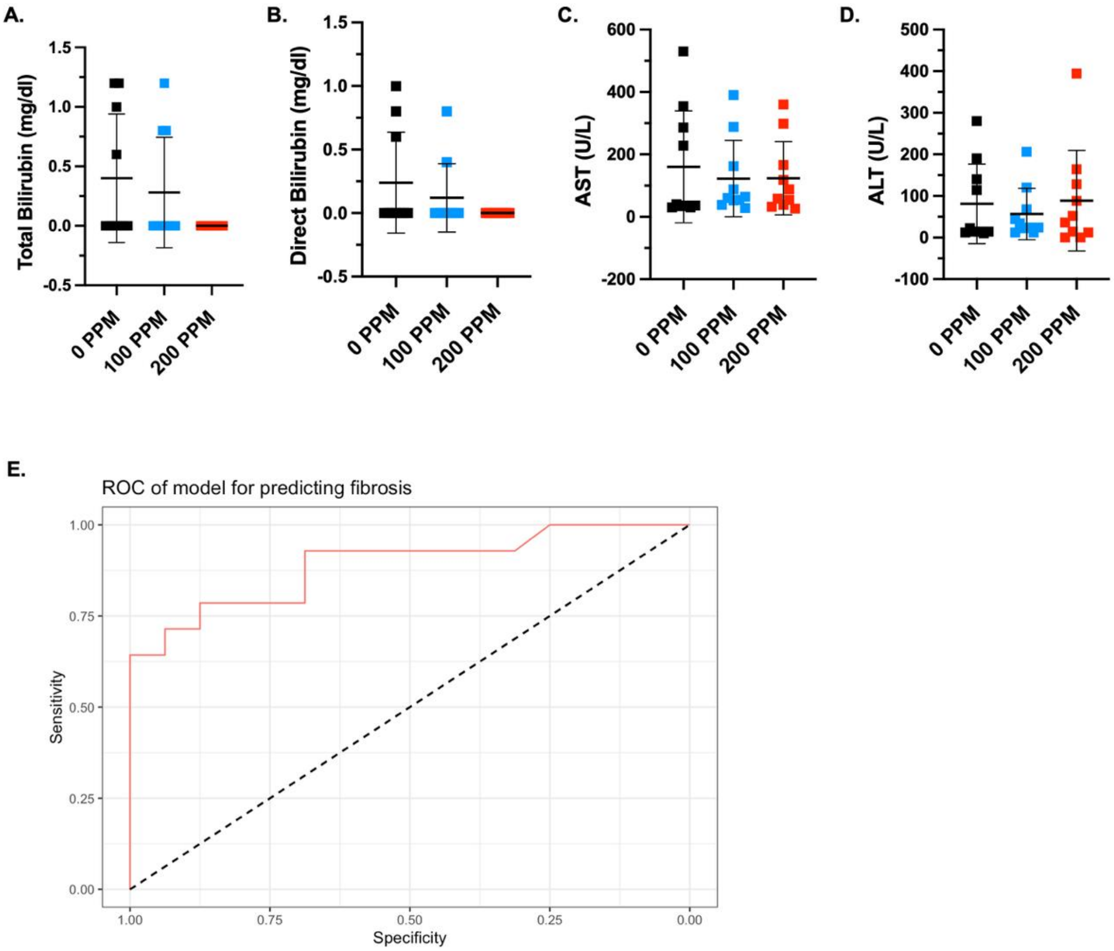

Supplemental Figure S6. Supporting data for Figures 9 and 10. EPP (Fech$^{-/-}$) male animals were fed a control diet (black), 100 ppm bitopertin (blue), or 200 ppm bitopertin for 8 weeks beginning at age 8 weeks. **(A)** Total bilirubin, **(B)** direct bilirubin, **(C)** aspartate aminotransferase, and **(D)** alanine aminotransferase were measured in serum at the end of the experiment. *P*-values were calculated using a Kruskal-Wallis test with Dunn's multiple comparisons test. **(E)** Receiving Operating Curve (ROC) of model for predicting fibrosis. The logistic regression model for this curve includes the variables of whether the mice were on bitopertin and the baseline MFI. The area under the receiver operating curve (AUC) is displayed, which reflects this model's ability to predict fibrosis in the current dataset.

Supplemental Figure 7 (EPP 40w)

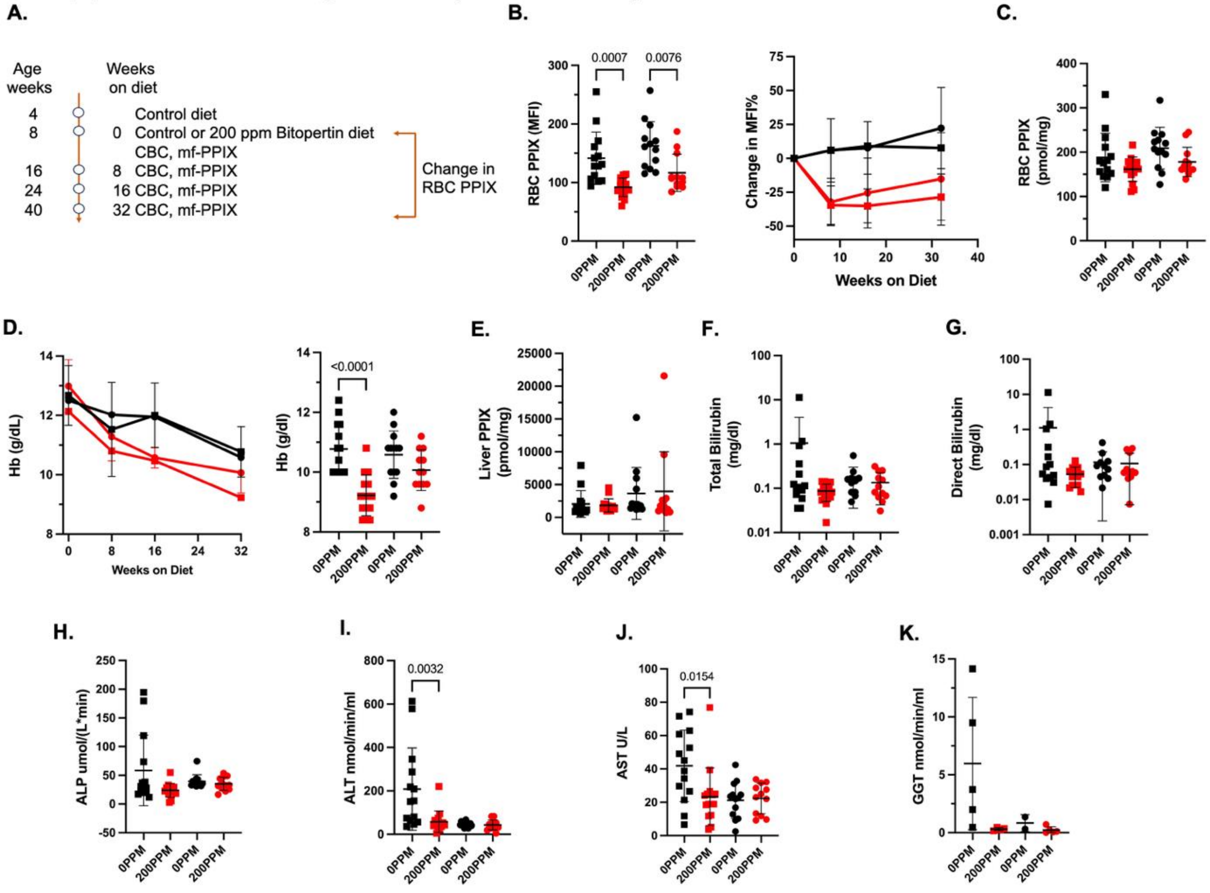

L.

Number of EPP animals	0 ppm Male	200 ppm Male	0 ppm Female	200 ppm Female
Enrolled	16	15	16	15
At Euthanasia	14	15	13	12

M.

	study set						
	14W		16W			40W	
Bitopertin (concentration)	0 ppm	100 ppm	0 ppm	100 ppm	200 ppm	0 ppm	200 ppm
incidence of hepatocellular carcinoma (HCC)	0/17	0/18	0/10	0/10	0/10	1/28	0/27
incidence of dysplastic nodule	0/17	0/18	0/10	0/10	0/10	3/28	0/27

N.

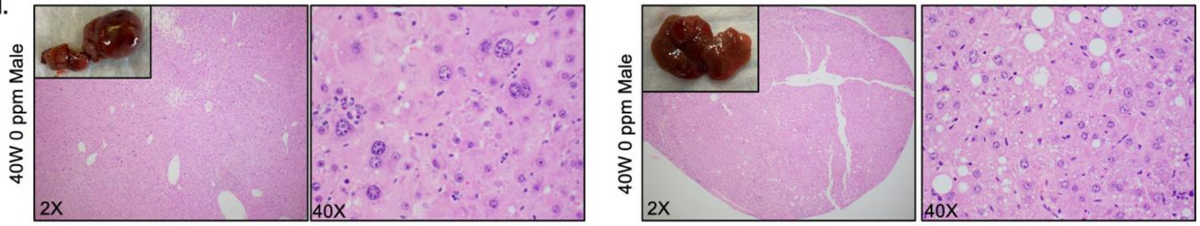

Hepatocellular carcinoma

Dysplastic nodule

Supplemental Figure S7. Supporting data for Figure 11 and Tables 1 and 2. EPP ($Fech^{-/-}$) male (square) and female (circle) animals were fed a control diet (black) or 200 ppm bitopertin (red) for 32 weeks beginning at age 8 weeks. **(A)** Treatment and sampling schema. **(B)** RBC mf-PPIX MFI at 40 weeks (left) and change in over time (right). **(C)** Quantitative RBC PPIX at takedown. **(D)** Hemoglobin (Hb) over the treatment time course (left) and after 40 weeks on treatment (right). **(E)** Quantitative liver PPIX after 32 weeks on bitopertin. **(F)** Total bilirubin, **(G)** direct bilirubin, **(H)** alkaline phosphatase, **(I)** alanine aminotransferase **(J)** aspartate aminotransferase, and **(K)** gamma-glutamyl transferase were measured in serum at the end of the experiment. P-values were calculated using a Kruskal-Wallis test with Dunn's multiple comparisons test. **(L)** Two male animals on control diet were euthanized by veterinary staff after fighting or self-mutilation, two female animals on control diet, and one on 200ppm diet, were euthanized by veterinary staff for severe dermatitis. One female on 200ppm diet was euthanized by veterinary staff for an eye issue. Due to a study duration adjustment proposed by the funder, two animals reaching week 24 - one female on a control diet and one female on a 200 ppm diet - were euthanized. Upon further consideration, the protocol was returned to its original duration and the remaining mice continued treatment until the originally planned endpoint at 40 weeks of age. **(M)** Incidence of hepatocellular carcinoma (HCC) or dysplastic nodule (number of animals with disease pathology/total mice treated). **(N)** Histopathology (hematoxylin and eosin) of representative EPP animals (Original magnification X2 left, X40 right) with hepatocellular carcinoma HCC (top) and dysplastic nodule (bottom).
