# Supplementary material for: The GLYT1 inhibitor bitopertin mitigates erythroid PPIX production and liver disease in erythroid protoporphyria
Source: J Clin Invest. 2025 Jul 15;135(18):e181875. doi: 10.1172/JCI181875 (PMC12435834; doi:10.1172/JCI181875)
Supplement: Unedited blot and gel images [file jci-135-181875-s046.pdf]

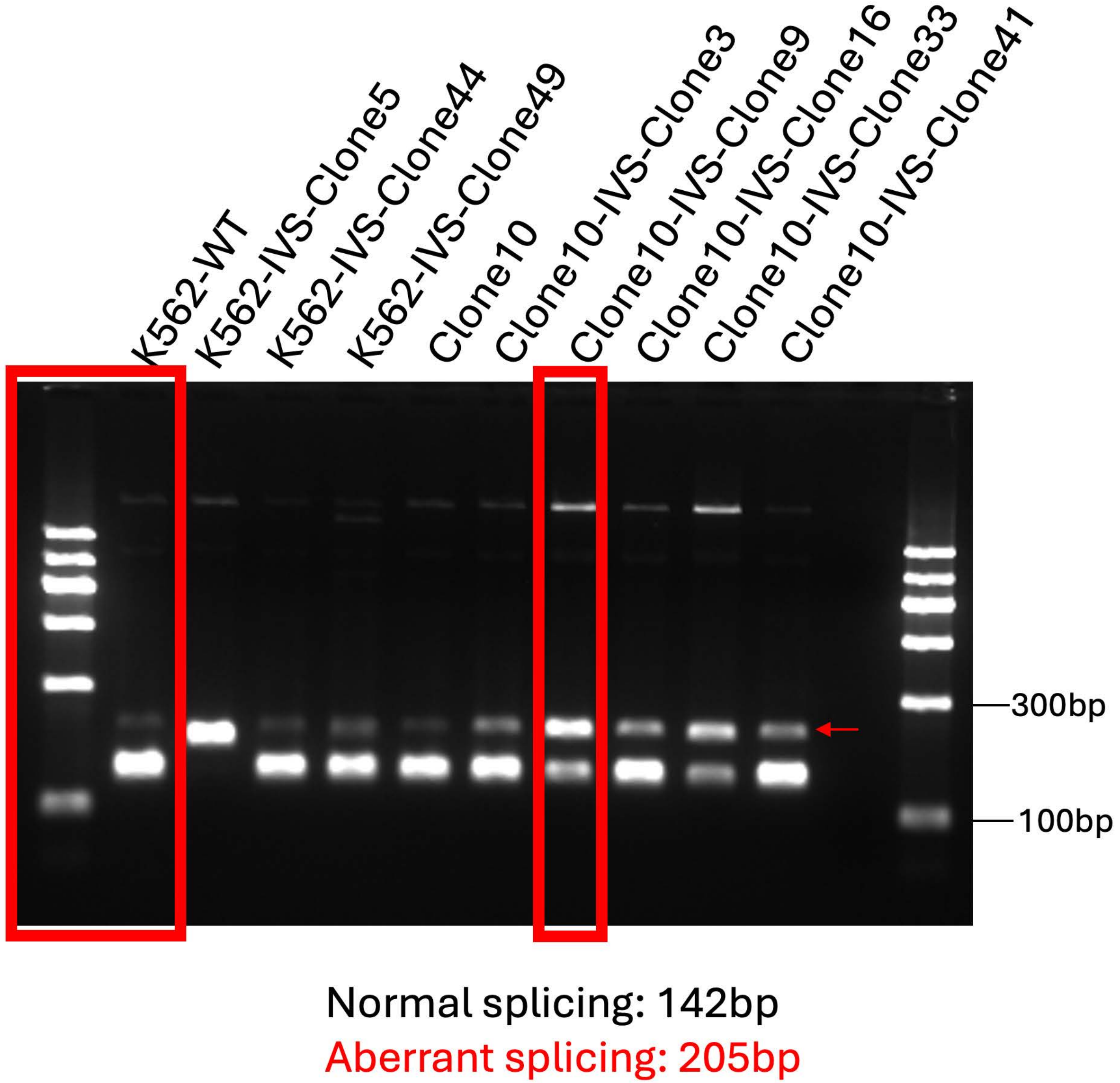

Full unedited blot/gel for Figure 1B
Lanes employed are highlighted in red

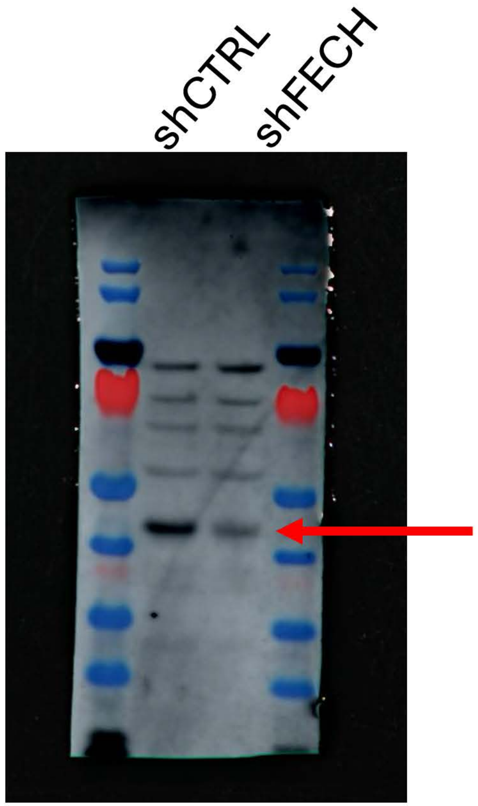

FECH, 14466-1-AP,
Proteintech

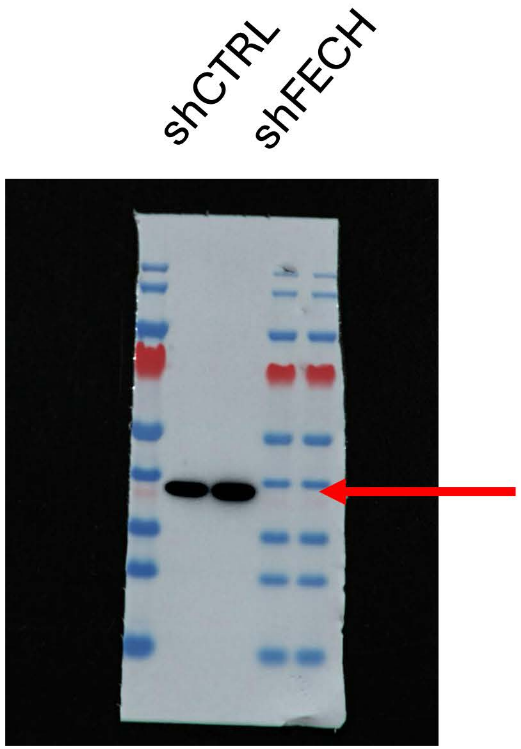

GAPDH, D16H11 XP® Rabbit mAb,
Cell Signaling Technology

Full unedited blot/gel for Supplemental Figure 1C
Bands employed are highlighted with red arrow
